# Biological and Biomedical Applications of Optical Photothermal Infrared Spectroscopy (O-PTIR)

**DOI:** 10.1177/00037028261447532

**Published:** 2026-05-16

**Authors:** Twinkle Soni, Diana E. Bedolla, Bayden R. Wood

**Affiliations:** 1 2541 Centre for Biospectroscopy, School of Chemistry, Monash University, Clayton, Victoria 3800, Australia; 2 550473 IIT-B Monash Research Academy, Indian Institute of Technology Bombay, Mumbai 400076, Maharashtra, India; 3 Advanced Light Source Division, 1666 Lawrence Berkley National Laboratory, Berkley, California, 94720 USA

**Keywords:** Optical photothermal infrared, O-PTIR, IR spectroscopy, Raman spectroscopy, fluorescence, sub-micrometer

## Abstract

Optical photothermal infrared (O-PTIR) spectroscopy is rapidly transforming molecular imaging by combining the chemical specificity of infrared absorption with the high spatial resolution enabled by visible-light excitation. This review provides a comprehensive and forward-looking overview of O-PTIR, with a particular focus on its expanding applications in biological and biomedical research. We begin by outlining the development of O-PTIR from earlier photoacoustic and photothermal infrared methodologies, placing it within the broader field of vibrational spectroscopy and emphasizing its unique advantages including sub-micrometer spatial resolution, label-free detection, and compatibility with heterogeneous biological samples. The foundational principles of O-PTIR, such as the photothermal effect, instrumental configurations, and the integration of simultaneous IR and Raman measurements, are discussed to establish the basis for its analytical capabilities. Leveraging these strengths, O-PTIR enables high-resolution and chemically specific imaging of key biomolecules including lipids, proteins, nucleic acids, and metabolites across cells, tissues, and microbial systems. Current applications span diverse areas such as cellular metabolism, microbial phenotyping, cancer diagnostics, biomarker identification, and pharmaceutical analysis. Alongside these advances, we critically evaluate existing limitations, including challenges associated with sample preparation, instrumental complexity, signal interpretation, and standardization. Finally, we highlight emerging directions such as live-cell O-PTIR measurements, artificial intelligence (AI)-driven spectral analysis, and the development of hybrid modalities including FISH-O-PTIR. Together, these innovations reinforce O-PTIR's potential as a transformative technology for biological and biomedical sciences, poised to bridge fundamental molecular studies with future clinical and translational applications.

## Graphical abstract

**Graphical abstract:**
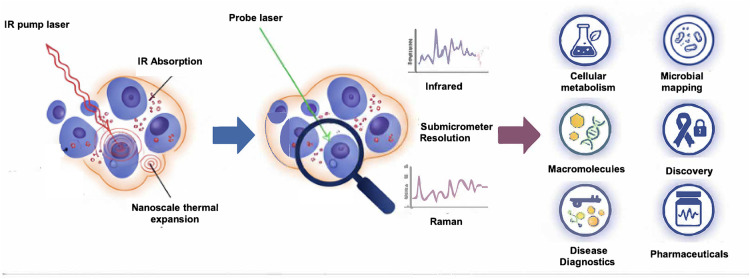
This is a visual representation of the abstract.

## Introduction

Understanding the complex molecular landscape of biological systems requires analytical techniques that are both chemically specific and spatially precise. Over the past few decades, vibrational and fluorescence-based spectroscopies have emerged as essential tools in biological and biomedical research. Among them, infrared (IR) spectroscopy, Raman spectroscopy, and fluorescence microscopy each provide unique insights into biomolecular structure, function, and interactions. However, the limitations inherent in each method have driven the development of advanced hybrid techniques such as, optical photothermal infrared (O-PTIR) spectroscopy, a powerful and versatile approach that combines the strengths of multiple modalities.^1–5^

Infrared spectroscopy has long been valued for its ability to identify molecular functional groups based on their characteristic vibrational absorptions in the mid-infrared region. Its non-destructive nature, broad applicability, and label-free operation make it especially suitable for probing the chemical composition of biological tissues, cells, and biofluids. However, IR spectroscopy suffers from a critical drawback limited spatial resolution due to the long wavelengths of mid-IR radiation.^6^ This diffraction limit typically restricts resolution to the order of several micrometers, which is inadequate for resolving subcellular structures or fine molecular distributions.

Raman spectroscopy, which measures inelastic scattering of light, offers complementary vibrational information and is particularly useful for detecting symmetric or non-polar bonds that are less prominent in IR spectra. Raman techniques are capable of sub-micrometer spatial resolution due to the use of visible excitation sources, and they can be implemented through confocal microscopy. Yet, Raman signals are inherently weak, often requiring long acquisition times or high laser powers that may damage biological samples. Furthermore, fluorescence background from endogenous chromophores can obscure the Raman signal, complicating data interpretation.

Fluorescence spectroscopy and microscopy, in contrast, provide excellent sensitivity and high spatial and temporal resolution. Through targeted labelling, fluorescence techniques can localize specific biomolecules or track dynamic cellular processes in real time. However, their reliance on external fluorescent tags poses several limitations, including potential perturbation of native biological functions, photobleaching, and spectral overlap in multiplexed assays. Moreover, fluorescence imaging lacks inherent chemical specificity, as it depends on the availability and selectivity of labels rather than the direct detection of molecular vibrations. Given these limitations, there is a growing need for techniques that combine the label-free chemical specificity of IR spectroscopy, the spatial resolution of Raman, and the imaging capabilities of fluorescence, while minimizing the respective drawbacks.

This review aims to provide a comprehensive overview of O-PTIR and its growing role in life sciences. The paper begins with an in-depth look at the principles and historical evolution of the technique, followed by a discussion on its significance relative to IR and Raman methods in biology. The unique advantages of O-PTIR are then highlighted, including its capacity for multimodal analysis, high-resolution imaging, and compatibility with complex biological samples. Subsequent sections dive into the underlying photothermal mechanisms, instrumentation, and experimental configurations, comparisons with conventional techniques and the expanding range of biological applications, from subcellular imaging and microbial analysis to disease diagnostics and pharmaceutical research. The review concludes with a discussion of current limitations, future directions, and the transformative potential of O-PTIR in addressing key challenges in biological and biomedical spectroscopy.

### Overview of Optical Photothermal Infrared Spectroscopy (O-PTIR)

Optical photothermal infrared (O-PTIR) spectroscopy has rapidly established itself as a pivotal technique in the realm of vibrational spectroscopy. By uniquely combining molecular specificity, high spatial resolution, and label-free imaging, O-PTIR addresses the growing demand in scientific research for methods that can interrogate the chemical composition of complex biological systems with minimal sample preparation. This technique effectively bridges the long-standing gap between conventional IR spectroscopy and advanced optical imaging, enabling chemical visualization at sub-micrometer scales. Consequently, it provides unprecedented insights into the structural and molecular features of cells, tissues, and materials.

At the heart of O-PTIR lies the photothermal effect, a phenomenon in which modulated mid-infrared radiation is absorbed by the sample, leading to localized heating. This thermal excitation alters the refractive index or causes minute thermal expansion in the sample, which is subsequently detected by a co-aligned visible probe laser. Since the detection relies on visible light, O-PTIR circumvents the spatial limitations imposed by the longer wavelengths of mid-IR radiation. This results in a spatial resolution that can exceed that of traditional Fourier transform infrared (FT-IR) spectroscopy by up to 30-fold.^2^ Such enhancement in resolution is particularly valuable in biological applications where fine structural distinctions and precise chemical mapping are essential.

In contrast to traditional IR methods, which often necessitate thick, uniform samples and typically deliver lower resolution, O-PTIR is adept at analyzing thin, heterogeneous specimens^7^ and even living cells with remarkable clarity.^8^ This capability is achieved without the need for external labels, such as fluorescent or radioactive tags, thereby maintaining the sample's native biochemical state and reducing the risk of measurement artifacts. The label-free nature of O-PTIR makes it especially well-suited for observing dynamic biological phenomena in real time, including metabolic shifts, protein conformational changes, and drug–biomolecule interactions.

The evolution of O-PTIR has been built on foundations laid by earlier techniques like photoacoustic infrared spectroscopy, which utilized sound waves generated through IR absorption to gain molecular insights.^9–11^ Although informative, such methods were inherently limited by their spatial resolution and sensitivity. O-PTIR overcomes these limitations by employing advanced laser systems, high-sensitivity detectors, and precise optical configurations, yielding a robust platform for high-resolution and multimodal imaging. Beyond standard IR absorption, modern O-PTIR systems can integrate complementary techniques such as Raman spectroscopy, fluorescence microscopy, and polarization-sensitive imaging collectively offering a multidimensional chemical and structural view of the sample.

A particularly powerful feature of O-PTIR is its ability to collect both IR and Raman spectra from the same region of a sample, facilitating a dual-modality approach to molecular characterization. This combined methodology provides orthogonal and complementary vibrational information: IR spectroscopy is responsive to changes in dipole moments and is particularly effective for identifying polar functional groups, while Raman spectroscopy is sensitive to changes in polarizability and excels in detecting non-polar bonds. The synergy of these modalities allows for a complete and more nuanced molecular fingerprint of biological materials, thus enabling more accurate and holistic biochemical analysis.

**Figure 1.**
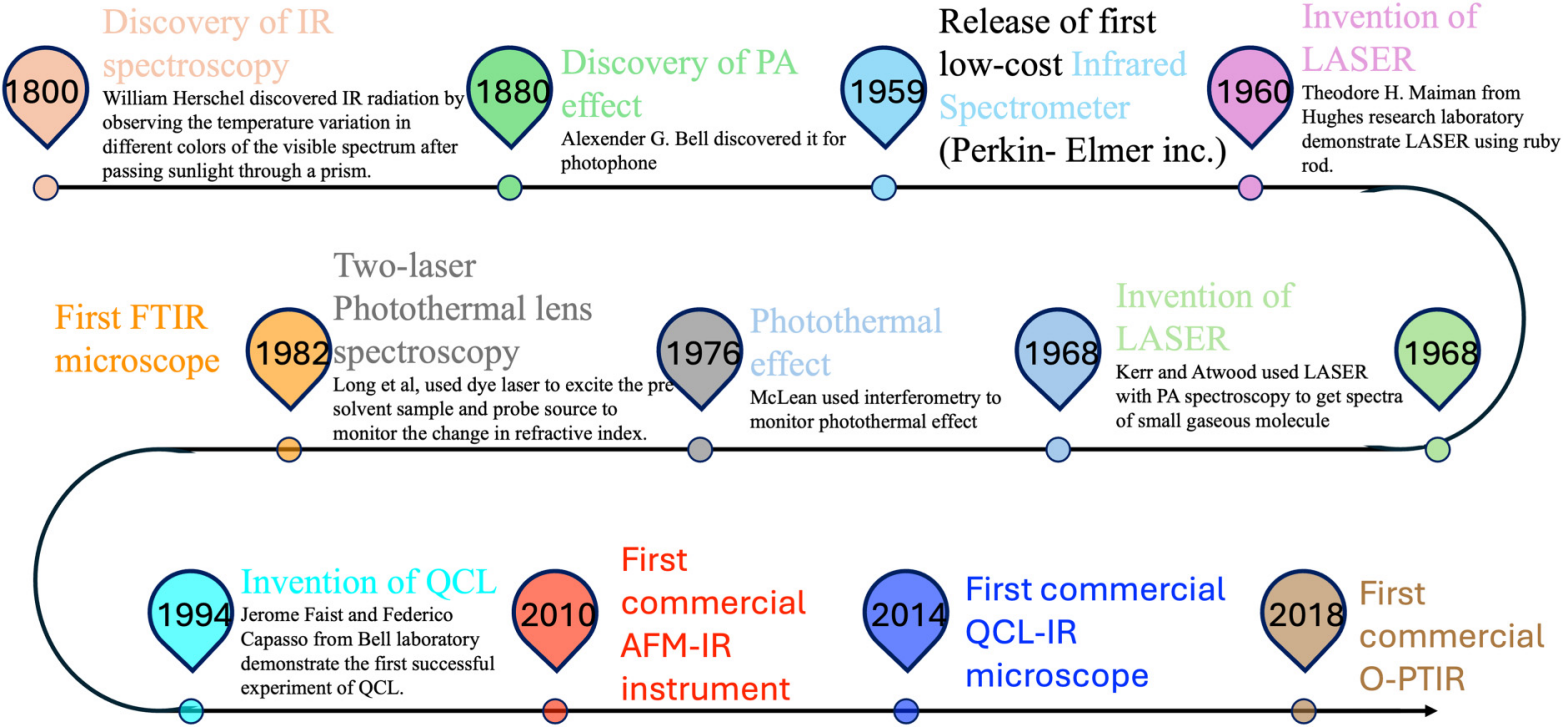
Historical development of O-PTIR from discovery of IR spectroscopy in 1800.

Having outlined the capabilities of O-PTIR, it becomes clear why this technique has obtained significant attention across biological and biomedical disciplines. By integrating the chemical specificity of infrared absorption with the spatial precision of optical detection, O-PTIR offers a unique platform for high-resolution, label-free molecular imaging. However, to fully appreciate the innovation and impact of this technique, it is essential to understand the technological evolution that led to its development. The origins of O-PTIR can be traced back to earlier photothermal and photoacoustic methods, which laid the groundwork for the current advances in hybrid infrared spectroscopy. The next section explores this historical trajectory, highlighting the key milestones and scientific breakthroughs that culminated in the emergence of O-PTIR (Figure 1) as a cutting-edge analytical tool.

### Historical Development of O-PTIR: From Photoacoustic IR to O-PTIR

The story of the photothermal effect's first technical application begins in the late 19th century,^12^ with a remarkable invention by none other than Alexander Graham Bell. Though best known for inventing the telephone, Bell also created a lesser known yet visionary device—the photophone. In 1880–1881, Bell discovered that sound could be transmitted through light.^13,14^ When he shone modulated light onto a transparent tube filled with various substances, an unexpected phenomenon occurred the tube “spoke”. To Bell's amazement, the sound was loud when the tube contained radiation-absorbing gases or solids, but much fainter when filled with a liquid. At the time, the mechanism behind this acoustic response was not fully understood. Today, we know it was an early demonstration of the photothermal effect in action. As the light's intensity varied, the absorbing materials experienced corresponding temperature changes. In the confined space of the tube, these fluctuations led to rhythmic expansions and contractions of the gas essentially creating sound waves. What Bell had inadvertently built was a primitive version of what we now call photoacoustic spectroscopy.

Decades later, in 1938, a researcher named Viengerov^7^ took this effect a step further. He used modulated light to study the absorption of gases and, for the first time, linked the strength of the resulting acoustic signal to the concentration of gases in a mixture. Without realizing it, Viengerov had performed the earliest quantitative photoacoustic measurement laying the groundwork for a powerful analytical technique.

The field remained relatively quiet until the 1960s, when scientists began to revisit the idea, this time with laser beams of light far more intense and coherent than anything Bell or Viengerov had access to. Kerr and Atwood, in 1968, used lasers to excite samples, dramatically increasing the sensitivity of their measurements.^10^ Kreuzer demonstrated that with a helium–neon laser emitting at 3.39  µm, it was possible to detect methane in nitrogen down to parts-per-billion (ppb).^11^ Just a year later, Kreuzer, along with Kenyon and Patel, achieved sub-ppb detection of ammonia and other gases using infrared CO and CO_2_ lasers.^15^

These breakthroughs were more than just technical milestones; they came at a time when the world was beginning to realize that even tiny traces of gases could have major implications for environmental health. The incredible sensitivity of photoacoustic spectroscopy made it a timely tool for monitoring pollutants and trace species in air. As the method gained traction, theoretical foundations were laid by physicists like Herzfeld, Litovitz, Landau, and Lifshitz during the mid-20th century.^16,17^ They explored deep into the physics of sound generation, propagation, and interaction with matter offering mathematical models that helped explain and predict photoacoustic behavior. Though the theories were complex, and practical applications sometimes hard to interpret precisely, the core principles became widely accepted.

One of the earliest photothermal techniques applied for sensitive chemical analysis was photothermal spectroscopy. This effect was first identified by Gordon and colleagues in 1964,^18^ who noticed changes in both power and beam divergence when transparent samples were introduced into the cavity of a helium–neon laser. Their initial aim was to develop a high-intensity source for Raman spectroscopy, but unexpectedly, they observed that certain materials such as organic liquids and clear solids like glass or Lucite caused the laser beam to behave as though a lens had formed within the sample. This phenomenon was later explained through a theoretical model that described how localized absorption of laser energy leads to thermal gradients, which in turn alter the sample's refractive index. These changes cause the beam to converge or diverge, forming what is now known as the photothermal lens.^19^ The theoretical framework established at the time remains largely valid in current interpretations of the effect.

Following this discovery, Grabiner, Siebert, and Flynn^20^ were among the first to construct an external (extra-cavity) version of this setup, which they used to investigate vibrational relaxation processes. Their configuration enabled more flexible sample handling and improved the sensitivity of absorption measurements. Later refinements by Hu and Whinnery^21^ further demonstrated that this extra-cavity approach offered significant advantages for studying weakly absorbing systems, establishing photothermal lens spectroscopy as a robust tool for thermal and optical analysis.

Not long after the discovery of photothermal lens effect, scientists began exploring the new ways to measure the resulting optical changes more directly, photothermal-induced refractive index change could be measured by more direct means. McLean, Sica, and Glass^22^ and Longaker and Litvak^23^ recognized that optical absorption resulting in sample heating and subsequent changes in refractive index would cause a phase shift in light passing through the heated region. The optical phase shift can be detected with an interferometer. The use of an excitation laser to heat the sample while tracking the refractive index change was novel, however, the technique of measuring refractive index changes using optical interferometry was not. Laser excitation sources are the foundation of the majority of photothermal interferometry equipment. Both coherent and wideband incoherent sources could be employed, as demonstrated by Stone.^24,25^ Stone obtained the chlorobenzene absorption spectra using a modified Jamin interferometer equipment.

Prior to the discovery of the additional cavity single-laser approach, the two-laser photothermal lens apparatus was utilized. Grabiner, Siebert, and Flynn^20^ probed the photo-thermal lens created by a pulsed infrared laser using a helium–neon laser. They calculated the vibrational relaxation rate constants for methyl fluoride and methyl chloride using their two-laser photothermal lens device. The method was then applied by Siebert, Grabiner, and Flynn^26^ to investigate the relaxation of vibrationally stimulated CD_4_, SO_2_, and OCS. The photothermal lens signal's risetime was of interest. The vibrational relaxation rate constants were inferred from the risetime observations as a function of the increased gas pressure. For relaxation times longer than the acoustic-limited risetimes, the method proved to be highly satisfactory. The rate constants for vibrational relaxation showed good agreement with those derived from previous techniques. While not utilized in this study, Sibert et al.^26^ showed that visible detectors may be used to assess infrared absorption through the use of this photothermal lens technique. Short path length IR absorption investigations might subsequently benefit from this.

Although the photothermal effect was recognized long before, its analytical potential remained largely untapped, until Boccara, Fournier, and Badoz^27^ proved probing laser beam deflection in 1979, the analytical technique based on this principle, i.e., photothermal deflection spectroscopy, was inexplicably disregarded. The cornerstone of mid-infrared photothermal spectroscopy was laid by Fournier et al. in 1983,^28^ who were the first to show photothermal imaging using the mirage phenomenon. A mirage is an optical illusion caused by the bending of light in layers of air with different temperatures, making distant objects appear displaced or reflected.

Photothermal deflection can be used to study a heated surface by examining its time-dependent thermoelastic displacement under pulsed or continuous laser excitations, in addition to the thermally induced refractive index gradients in the solid material and the coupling fluid. We call this process photothermal displacement. The approach was introduced by Olmstead et al. in 1983^29^ as a sensitive way to determine the optical and thermal properties of solids, surfaces, and thin films. Karner, Mandel, and Träger^30^ examined transient heating events in real time using pulsed stimulation. Li^31^ introduced the three-dimensional theory of pulsed photothermal deformation for thin films, whereas Kuo and Munidasa^32^ employed a single-beam interferometric technique to detect the surface displacement of solids. The technique was applied by Bennis et al.^33^ to quantify the thermal diffusivity of materials. By explaining how the photo-displacement, the surrounding thermal lens, and the photoreflectance effects affect the displacement signal, Cheng and Zhang^34^ and Fang and Zhang^35^ employed this method to study semiconductors.

Fourier transform infrared photoacoustic spectroscopy (FT-IR-PAS) also known as photoacoustic spectroscopy, is a technique that relies on the absorption of infrared (IR) radiation by a solid sample. When IR light is absorbed, it causes slight thermal expansion of the sample, producing acoustic waves that are detected using an ultra-sensitive microphone, typically in a nitrogen environment. While FT-IR-PAS serves as a valuable accessory to traditional FT-IR, especially for analyzing bulk or otherwise difficult-to-handle samples, it historically lacked spatial resolution capabilities.^1^ Although photothermal imaging and spectroscopy have shown significant promise since the foundational work of Fournier,^28^ it took nearly three decades before researchers recognized that photothermal detection methods could be used to overcome the diffraction limit that constrains mid-infrared (mid-IR) spectroscopy. The first breakthroughs in surpassing the mid-IR diffraction limit came from atomic force microscopy (AFM).^36^ In these approaches, the AFM probe was used to sense the minute thermal expansions of a sample triggered by IR absorption enabling high-resolution, nanoscale infrared spectroscopy.

The initial research on the method's potential, which combines thermal microscopy and FT-IR spectroscopy, is cited in 1996.^37^ The probe was made of a 75 μm-diameter Wollaston filament that was looped and bent sharply to serve as a temperature sensor. A globar was the source of the infrared radiation. The sample and the probe were positioned in the beam's focus within a chamber of an FT-IR spectrometer that was manufactured in batches. In this instance, the probe records variations in the sample's surface temperature while functioning in a passive mode. Since the probe is in the beam's focus, it will inevitably be heated by the excitation beam as well as by contact with the sample surface, as is the case with probes of any other alternative temperature measuring device, such as thermocouples. This contributes to unacceptably high background levels. It is advised to record a contactless background spectrum, one in which the probe does not encounter the sample's surface and then subtract it from the sample's initial spectrum to remove the effect. The reversed conventional infrared spectra^38^ are quite similar to photothermal spectra. According to Bozec,^38^ who recorded photothermal spectra of powders, slight variations in the band shapes are caused by sample preparation. Additionally, the alterations could be explained by the fact that thermal variables other than optical ones can affect the generation of photothermal spectra, including the production, detection, and propagation. Because the linear relationship between the signal and the absorption coefficient is broken at high levels, photothermal spectra may show photothermal saturation. The peak suppression^38^ is the manifestation of this phenomenon.

One of the reasons for the low quality of the first photothermal spectra is the insufficient irradiance of the IR radiation. Replacing the globar with a more powerful source can significantly shorten the experimental time. Laser sources, for example, a 25 W CO_2_ laser^39^ or, more recently a quantum cascade laser (QCL), serve as alternatives. Pollock and Hammiche^40^ introduced a pulsed laser as a tunable source of IR radiation in this technique. Synchrotron IR radiation significantly improves the quality of the spectra.^41^ The application of scanning thermal microscopy (SThM) probes in photothermal infrared spectroscopy was documented by Hammiche et al. in 1999.^39^ Although there is a lot of noise in these spectra, the primary characteristic bands such as those that correspond to the valence C–H vibrations are easily discernible. This method's unquestionable benefit is how simple it is to clean the probe by transferring the sample material to the solution. A probe with a 150 × 50  nm palladium sensing element^42^ on the flat top of a small, truncated pyramid provides a greater resolution than that of SThM. One of the drawbacks of employing this kind of probe is that it has a lower signal irradiance than a SThM probe. Additional publications^37,43,44^ discussed a technique called near field photothermal FT-IR microspectroscopy, which combines the capabilities of FT-IR spectroscopy and an atomic force microscope. In this instance, the SThM probe is a component of an AFM microscope rather than a standard mobile stand. The authors have created a unique embedded AFM device design that can be installed straight into the chamber of the majority of FT-IR spectrometers.^45^

Thermal mirror and photothermal deflection techniques are somewhat linked to photothermal infrared microspectroscopy, also known as photothermal-induced resonance spectroscopy. Nonetheless, it originated from photothermal effects in AFM and was first focused on resolving issues with infrared.^46^ Instead of using the typical detector of FT-IR spectrometers,^37,47,48^ photothermal IR micro spectroscopy uses probes that are similar to those used in several scanning probe microscopic techniques, e.g., AFM, SThM, or scanning near-field optical microscopy (SNOM). Such a probe detects the transitory and local dilatation of a material upon resonance heating by a tunable infrared laser, which is the foundation of near field photothermal infrared microspectroscopy. When an absorption line of a sample constituent absorbs the excitation light, the photothermal signal rises.^49,50^ These probes enable the acquisition of spectra with a resolution as low as 20–30  nm, which is significantly higher than that of conventional IR spectroscopy. The resolution is solely dependent on the size of the micro-thermal probe and the thermal characteristics of the sample, not the wavelength of the IR radiation emitted by the source. Additionally, the resolution has an impact on the microthermal probe's thermal conductivity, the probe and sample's ultimate contact area, the system's complicated inhomogeneous temperature distribution, and the variation of the absorbed heat flux with respect to time and wavelength.

Though AFM-IR being a breakthrough technology, it comes with some drawbacks. The two configurations of AFM-IR, i.e., bottom up and top-down configuration are based on the illumination of tunable laser on the sample. One significant limitation of the bottom-up configuration lies in the maximum sample thickness that can be examined. Since this method relies on infrared evanescent wave illumination, the distance between the prism surface and the sample's top must remain under approximately 1 μm.^45^ Additionally, the sample material must be compatible with the properties of the prism, which restricts the types of samples that can be used. An alternative approach is the top-down illumination setup, which offers greater flexibility. This is widely used configuration in AFM-IR. This configuration removes constraints related to sample thickness and often eliminates the need for an infrared-transparent substrate. As a result, preparing samples becomes easier and a broader range of materials can be utilized. However, a major disadvantage is that the infrared laser beam directly hits the cantilever. To prevent absorption and enhance the field, the cantilever typically needs a metal coating, such as gold or platinum. This requirement narrows the selection of suitable AFM probes, and additional care must be taken to consider polarization effects when using these metal-coated tips.^36^ Another major drawback with AFM-IR is the requirement of high technical skills with high measurement time.^1^

These drawbacks spurred the development of alternative techniques that could overcome the diffraction limit of IR light, provide higher spatial resolution and quick in nature. The breakthrough came in 2012 with the use of visible wavelength optical probe beam to detect the IR absorption thus facilitating the beating of mid-IR diffraction limit.^51^ Sander et al. also presented a first compact all-fiber probe laser system for mid-IR photothermal spectroscopy in 2014. From same authors, they also used QCL to excite the sample and hence, reduced the time require to map the sample. This work led the foundation of the concept for high resolution infrared spectroscopy.^52,53^ Furthermore, this group demonstrated the potential of the technique to be used on biological samples such as tissues.

The transition from conventional FT-IR microscopy to O-PTIR represents a major paradigm shift in vibrational spectroscopy. While FT-IR and ATR microspectroscopy have served as powerful tools for decades, their spatial resolution has been fundamentally limited by the diffraction of mid-IR light, often exceeding several micrometers. In addition, sample preparation complexities especially for transmission mode and spectral artifacts have limited broader adoption in biological research. There have been a series of developments in the mid-IR approaching this photothermal effect with some variations.^6,^^54–59^ Some they have called this same technique VIPPS that stands for vibrational infrared photothermal amplitude and phase signal (VIPPS) imaging. In other works, the use of synchrotron radiation is used instead of the QCL source to obtain a broadband spectrum and compared using fluorescence-detected IR photothermal imaging,^60^ which takes advantage of the temperature dependence of fluorescence emission efficiency when compared to standard O-PTIR.^61^ However, this review will be mainly focused on the capabilities of the optical photothermal infrared spectroscopy technique from Photothermal Inc.

Optical photothermal infrared spectroscopy was developed to overcome these spatial resolution limitations by decoupling the resolution from IR wavelength. It utilizes a visible laser probe to detect photothermal responses induced by a tunable IR pump, enabling sub-micrometer resolution imaging (∼400  nm) and artifact-free spectra in reflection mode. Unlike ATR, O-PTIR is non-contact and does not require thin sample sectioning, simplifying sample handling across biological and materials science applications. This innovation, now commercially available through systems such as the mIRage, has opened new frontiers in spatially resolved spectroscopy, particularly for soft and fragile biological materials.

## Fundamental Principles of Optical Photothermal Infrared Spectroscopy (O-PTIR)

### The Photothermal Effect: A Detailed Explanation

**Figure 2.**
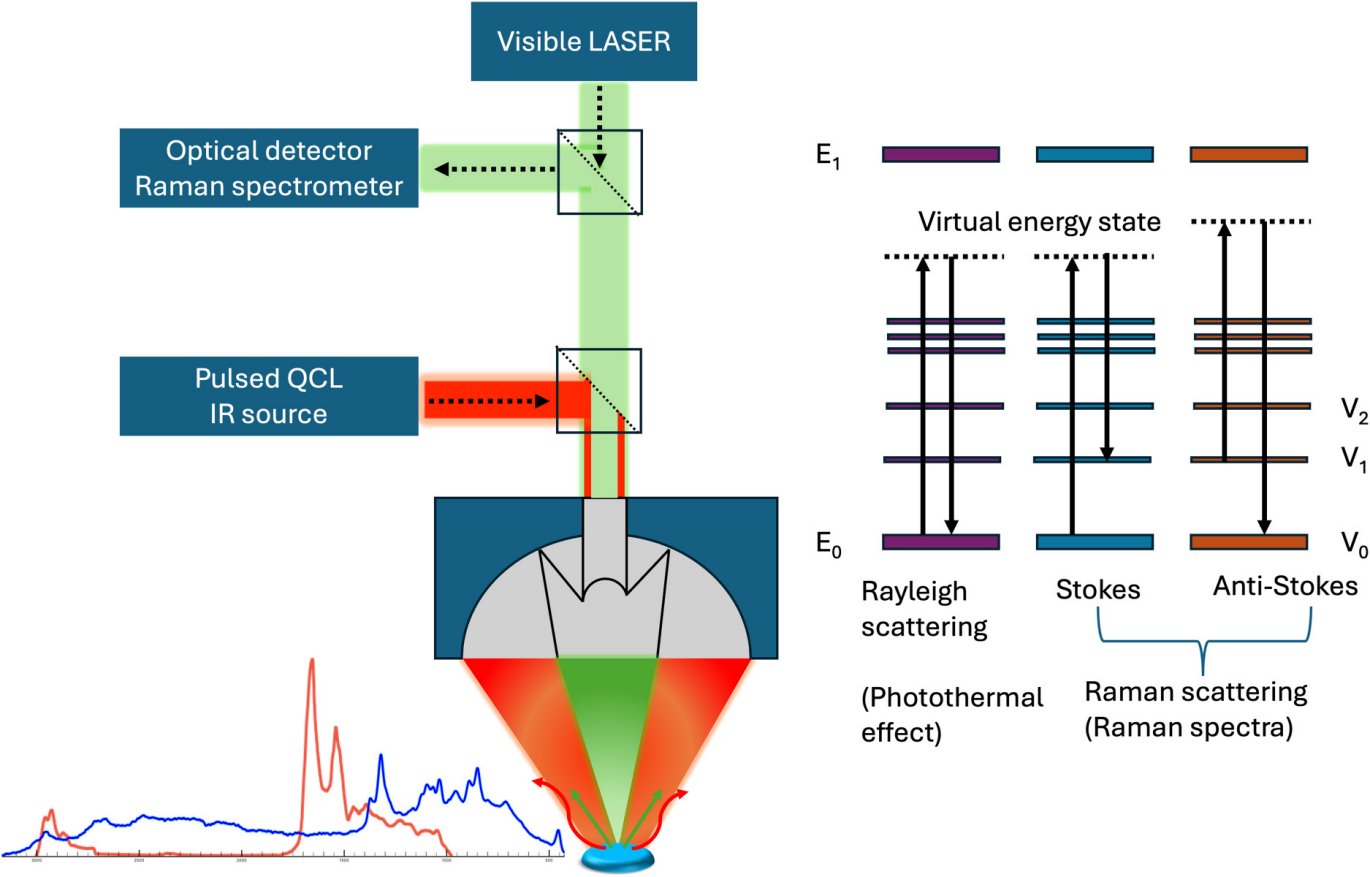
Schematic representation of working principle of O-PTIR.

Traditional optical spectroscopy techniques like ultraviolet–visible (UV–Vis) spectroscopy, fluorescence spectroscopy often rely on detecting absorption, fluorescence, or scattering signals. However, these methods face limitations when dealing with low-concentration analytes, opaque or turbid samples, or environments where direct optical detection is challenging. In such cases, the minimal changes in light intensity can be difficult to resolve, especially when absorption is weak or when strong background interference is present. The photothermal spectroscopy is a sub class of optical spectroscopy. This was developed to overcome these challenges by detecting the heat generated from light absorption rather than the light itself (Figure 2). The journey of photothermal spectroscopy begins with the photothermal effect.

The photothermal effect is something we encounter in everyday life, often without noticing it. A simple example is walking barefoot on a beach on a hot summer day when the sand feels unbearably hot. This happens because the sand absorbs sunlight and converts it into heat. As long as the absorbed energy exceeds the heat lost to the surrounding air or deeper layers, the temperature continues to rise until thermal equilibrium is reached.

A more striking and familiar example is the optical mirage seen on hot roads, where the surface appears to shimmer like water. This effect, which underpins several photothermal spectroscopy techniques, is not caused by reflection but by refraction due to temperature gradients in the air. Sun-heated asphalt warms the air just above it, making the air less dense and lowering its refractive index. Light traveling through this gradient bends upward, creating the illusion of a reflective surface.^60^ Although such phenomena were observed long before they were understood, only in the past century have their underlying principles been fully explained and exploited. Advances in laser technology and thermal modelling have transformed the photothermal effect from a curious visual illusion into a powerful analytical tool, now widely used in chemical and materials spectroscopy.

The photothermal effect happens when a sample surface is irradiated by a periodically modulated light source, the absorbed optical energy is converted into heat, resulting in a localized, time-dependent temperature oscillation. This periodic heating generates a thermal wave that propagates into the material, away from the surface. Unlike conventional mechanical or acoustic waves, thermal waves are governed by diffusive heat transport and are thus characterized by an exponential spatial decay rather than true wave-like oscillations.

In a homogeneous medium, the resulting thermal wave is critically damped, and the temperature amplitude decays exponentially with increasing depth from the heated surface.^11^ The characteristic length scale of this decay is described by the thermal wave damping distance, denoted as μ. This distance corresponds to the depth at which the temperature amplitude falls to 1/e (approximately 37%) of its surface value and is given by the expression:

(1) $$\mu =\sqrt{\left(\frac{2D}{\omega }\right)}$$

where D is the thermal diffusivity of the material (in m^2^/s) and ω is the angular modulation frequency of the excitation beam (in rad/s).

This relationship highlights that higher modulation frequencies result in more surface-confined heating, while lower frequencies allow for greater thermal penetration depths. This frequency-dependent depth control is a fundamental feature leveraged in photothermal and optothermal techniques for depth-sensitive analysis.

Alternatively, in the case of impulse heating, such as that induced by a short laser pulse, the heat propagates diffusively from the surface into the sample with a depth-dependent, Gaussian-like temperature profile.^62^ The characteristic depth of thermal penetration, μ_l_, at a given time *t*, is defined as:

(2) $$\mu_{l}=\sqrt{\left(2Dt\right)}$$

Here, the heat propagation is purely governed by time and thermal diffusivity, with the temperature front broadening as a function of time after the pulse. This time-dependent spreading of heat provides a distinct temporal resolution, enabling dynamic thermal profiling of materials following transient excitation. Together, both modulated and impulsive heating modes underpin a wide range of photothermal measurement techniques by enabling controlled spatial confinement of heat and allowing for subsurface probing with tunable thermal penetration depths.

Photothermal methods enable depth-resolved measurements of thermal and spectroscopic properties in condensed-phase samples.^62^ This is possible because the sampling depth of the technique can be controlled by either: Adjusting the modulation frequency of the excitation light (as described in Eq. 1) or changing the observation time following a pulsed excitation (as described in Eq. 2).

In techniques that monitor surface temperature changes, it is essential that the heat generation occurs within the thermal diffusion length (or damping distance) of the surface to influence the detected signal. When the thermal penetration depth μ increases (e.g., by lowering modulation frequency), deeper layers of the sample contribute more strongly to the signal. This alters the measured absorption spectrum, especially in optically transparent or layered materials. Photothermal spectroscopy can therefore be used for depth profiling, which may involve thermal profiling understanding how heat flows through different layers and/or spectroscopic profiling identifying changes in chemical composition with depth.

Although all derive from the same fundamental photothermal effect, techniques like photoacoustic spectroscopy, infrared (IR) radiometry, and photothermal spectroscopy are often discussed as distinct methods.^12,36^ Photothermal spectroscopy typically involves monitoring temperature-induced changes in the refractive index, often using a probe laser. However, photoacoustic signals pressure waves generated by periodic heating are inherently linked to the same underlying physics and cannot be entirely separated from photothermal spectroscopy, especially when considering hydrodynamic relaxation. In this context, the rate at which the sample returns to isobaric conditions influences the observed response. Additionally, IR emission represents another pathway for thermal energy dissipation and should be accounted for when evaluating the overall photothermal signal. A thorough understanding of the photothermal effect must consider all these interconnected processes.

### Instrumentation and Experimental Setup

The performance of O-PTIR spectroscopy of Photothermal Inc. is governed by beam geometry, focusing optics, and detection configuration, with two primary modes employed, co-propagation and counter-propagation. Each configuration presents distinct trade-offs in numerical aperture, spatial resolution, sample compatibility, and optical throughput, with recent advances incorporating fluorescence-guided targeting to improve acquisition efficiency.

**Figure 3.**
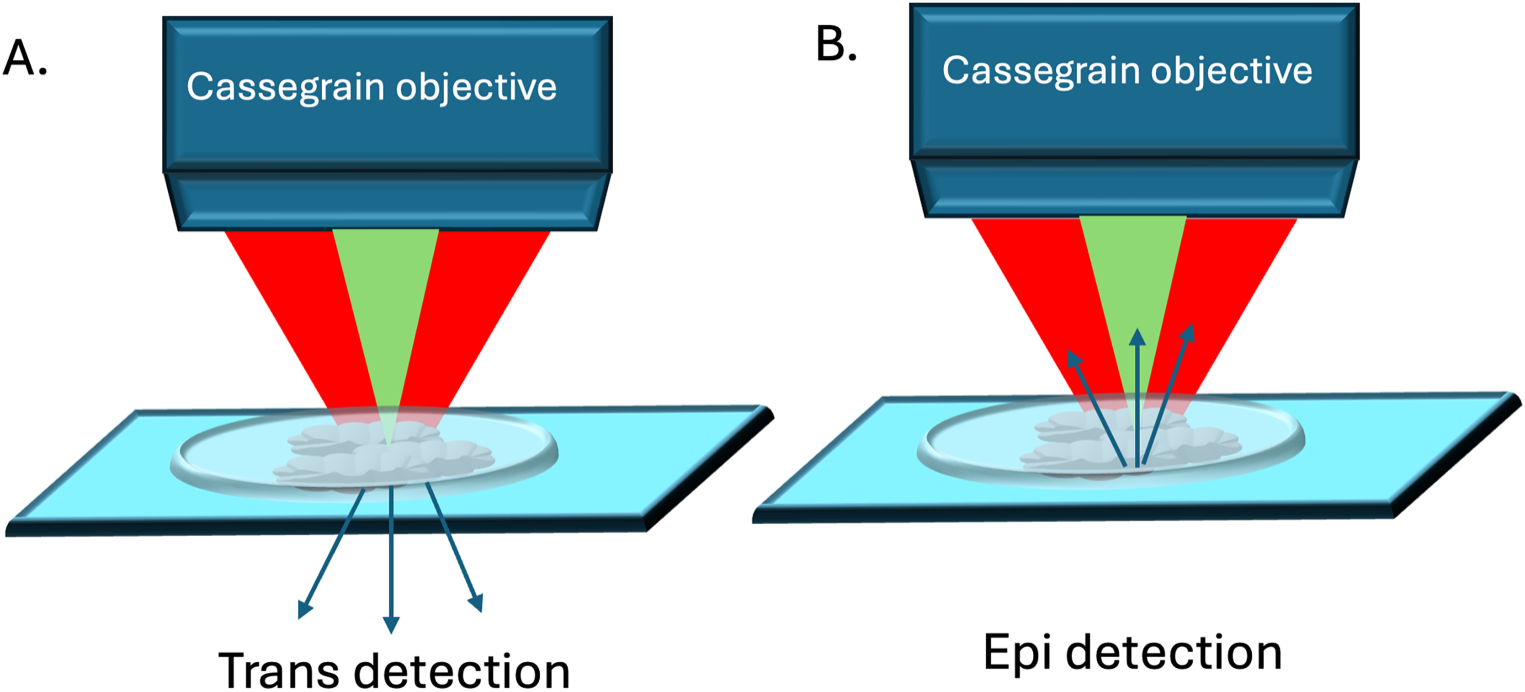
Different mode of collection associated with co-propagation mode of O-PTIR.

In the co-propagation configuration, pulsed mid-IR radiation from a tunable QCL and a visible probe beam (typically 532 or 785  nm) are delivered collinearly through a reflective Cassegrain objective.^1^ While the IR spot size remains diffraction-limited (≈5–10  µm), the visible probe defines the effective spatial resolution, achieving sub-micrometer resolution (∼0.4–0.8 µm^63^). This configuration supports flexible substrate choice and is well suited for opaque or thick samples, offering robust signal collection with controlled IR and probe power levels using silicon photodiodes or avalanche photodiodes. Transmission detectors are sometimes employed for samples with low reflectivity or for liquid specimens, though they are generally less optimal in co-propagation configurations due to reduced signal strength and increased complexity.^64–66^ Figure 3 shows different modes with co-propagation, trans detection, and epi detection.

In contrast, the counter-propagation configuration delivers the IR beam through an IR-transparent substrate from below, while the visible probe is focused from above using a high numeric aperture (NA) objective (up to ∼1.4).^67–69^ This decoupled geometry enables tighter visible focusing (∼200–300  nm) and improved spatial resolution (∼250–400  nm), approaching the diffraction limit of the probe beam. However, it requires thin, flat samples (≤10  µm) and IR-transparent substrates, and demands careful power optimization to avoid photothermal or photodamage artifacts. Table I compares the different modalities available with co and counter propagation.

**Table I.** Characteristic features associated with co-propagation and counter propagation configuration of the commercially available O-PTIR.

Parameter	Co-propagation	Counter propagation
IR wavelength range	2.5–12 µm (4000–833 cm^−1^)	2.5–12 µm (through substrate)
Visible probe wavelengths	532 or 785 nm typical	532 or 785 nm typical
IR spot size	5–10 µm	5–10 µm
Visible spot size	400–800 nm	200–300 nm
Spatial resolution	0.4–0.8 µm^63^	0.25–0.4 µm
IR power at sample	0.1–5 mW	0.05–3 mW
Visible power at sample	0.5–5 mW (PIN), 1–100 µW^70,71^ (adaptive peak detection, APD)	0.1–1 mW typical
Sample thickness	No strict limit^72^	≤10 µm
Substrate requirement	None^3,5,72^	IR-transparent
Best for	Thick/opaque specimens, opaque substrates^72^	Thin, transparent specimens, high resolution

## Fluorescence-Guided O-PTIR

Fluorescence imaging is a cornerstone of modern life sciences, enabling researchers to visualize specific cellular or tissue components with high specificity using fluorescent dyes, immunofluorescent staining, or genetically encoded fluorescent proteins. While these methods excel at pinpointing particular biological structures, they provide limited information about the broader molecular composition or the chemical structure of the labelled targets.^73,74^ O-PTIR spectroscopy offers a powerful, complementary approach by enabling direct, label-free probing of molecular structures and conformations at sub-micrometer spatial resolution.^2^ When combined with fluorescence imaging, O-PTIR can leverage fluorescence labels or even intrinsic autofluorescence as spatial landmarks, guiding targeted chemical analysis to regions of interest. This integrated strategy merges the targeted precision of fluorescence with the rich, label-free molecular insight of O-PTIR, enabling applications ranging from secondary structure analysis of proteins in disease-related aggregates to rapid hyperspectral mapping of complex biological specimens.

In fluorescence imaging, excitation wavelengths are typically selected based on the specific fluorophore requirements, with common choices including 405  nm, 488  nm, and 561  nm. Detection is carried out using high-sensitivity scientific complementary metal-oxide-semiconductor (sCMOS) cameras, which are well suited for capturing low-light signals with minimal noise. To reduce the risk of photobleaching and preserve sample integrity, the excitation power at the sample is usually maintained in the range of 10–200 µW. There are several advantages of fluorescence guided O-PTIR like it, identifies regions of interest (ROIs) before IR mapping, eliminates acquisition over irrelevant areas, reducing IR exposure and allows correlative mapping of fluorescence and IR for molecular and biochemical co-localization.

## Widefield Fluorescence in O-PTIR

Widefield fluorescence capitalizes on the significant temperature sensitivity of fluorescence emission rather than relying on subtle changes in refractive index. While conventional O-PTIR senses IR absorption through minute thermal deflections (∼10^–^^4^ per °C), fluorescence quantum yield changes by ∼1% per °C offering up to 100× higher sensitivity.^4,61^ This enhanced contrast enables rapid, wide-field detection of IR absorption by^75^ modulations in fluorescent intensity induced by local, IR-triggered temperature increases.

In widefield fluorescence a high sensitivity two-dimensional (2D) fluorescence camera (e.g., sCMOS) captures entire fields of view in parallel akin to IR focal plane arrays allowing simultaneous acquisition at hundreds of thousands of pixels. Typical arrays (e.g., 512 × 512 pixels, 130  nm per pixel) deliver fields of view around 65 × 65 µm^2^ per tile, with frame rates up to ∼5 fps for single-wavelength imaging and full hyperspectral stacks acquired within minutes.^1^ Spatial resolution reaches sub-500  nm,^76^ and in many cases hits sub-300  nm, leveraging the limits of fluorescence optics rather than IR diffraction. Applications include, biological samples^2^ (bacteria, live cancer cells, collagen), hyperspectral imaging, pharmaceutical materials, polymer^61^ and tissue mapping,^2^ etc.

Widefield O-PTIR enables rapid, large-area chemical and structural imaging, allowing efficient identification of regions of interest for high-resolution or correlative studies. Hyperspectral snapshot imaging provides sub-micrometer chemical maps while significantly reducing acquisition time and photodamage, and fluorescence detection can enhance sensitivity by up to two orders of magnitude, benefiting low-absorbing and dynamic biological samples. However, performance is limited by illumination uniformity, camera pixel size, objective NA, and IR power density, often requiring mosaicking, while fluorescence-enhanced O-PTIR also demands careful control of photobleaching, photostability, and spectral compatibility for reliable measurements.

## Polarization-Sensitive O-PTIR

A unique strength of O-PTIR lies in the high polarization purity of QCL-based IR sources, enabling molecular orientation analysis in polymers, biomaterials, and complex tissues. The efficiency of IR absorption by a molecular vibration depends on the alignment between the bond dipole axis and the electric field vector of the incident IR beam.^2^

By acquiring O-PTIR spectra or images at multiple polarization orientations commonly at least three distinct angles the principal molecular orientation can be deduced without ambiguity. Early studies achieved this by physically rotating the sample, but modern instruments integrate polarization rotation modules to electronically control the IR polarization, avoiding sample repositioning. Applications includes, biopolymers,^77,78^ tissue analysis,^78^ polymer crystallinity,^64,79^ etc. For such measurements, careful control of IR power (∼10–500 µW at the sample for sensitive materials) and beam shape is critical to avoid thermal artifacts while maintaining orientation sensitivity.

## Single Wavenumber Scanning and Hyperspectral Imaging in O-PTIR

One of the versatile features of O-PTIR spectroscopy is its ability to operate in different acquisition modes that balance spectral information with spatial resolution and acquisition speed. Two commonly employed approaches, single wavenumber scanning and hyperspectral imaging offer complementary benefits for biological and material analysis.

### Single Wavenumber Scanning

In single wavenumber mode, the QCL source is tuned to a fixed wavenumber corresponding to a specific vibrational mode of interest (e.g., amide I, amide II, lipid CH stretching). The probe beam captures the photothermal response only at this frequency, enabling rapid acquisition of high-resolution spatial maps. One of the key advantages of this approach is its speed, as it allows for much faster data acquisition compared to collecting a full spectrum at every pixel. This efficiency is especially beneficial when rapid imaging is required. Additionally, the method reduces photodamage by minimizing the total infrared exposure to sensitive samples, helping to preserve their integrity during measurement.^71^ Furthermore, targeted analysis becomes possible and highly effective when the relevant spectral bands are already known from previous experiments, enabling focused investigation of specific chemical or structural features without unnecessary data collection. The application of single frequency scanning can be seen in visualizing protein or lipid distribution, detecting disease-specific markers, and monitoring dynamic processes in living cells where speed is critical.^80,81^

### Hyperspectral Imaging

**Figure 4.**
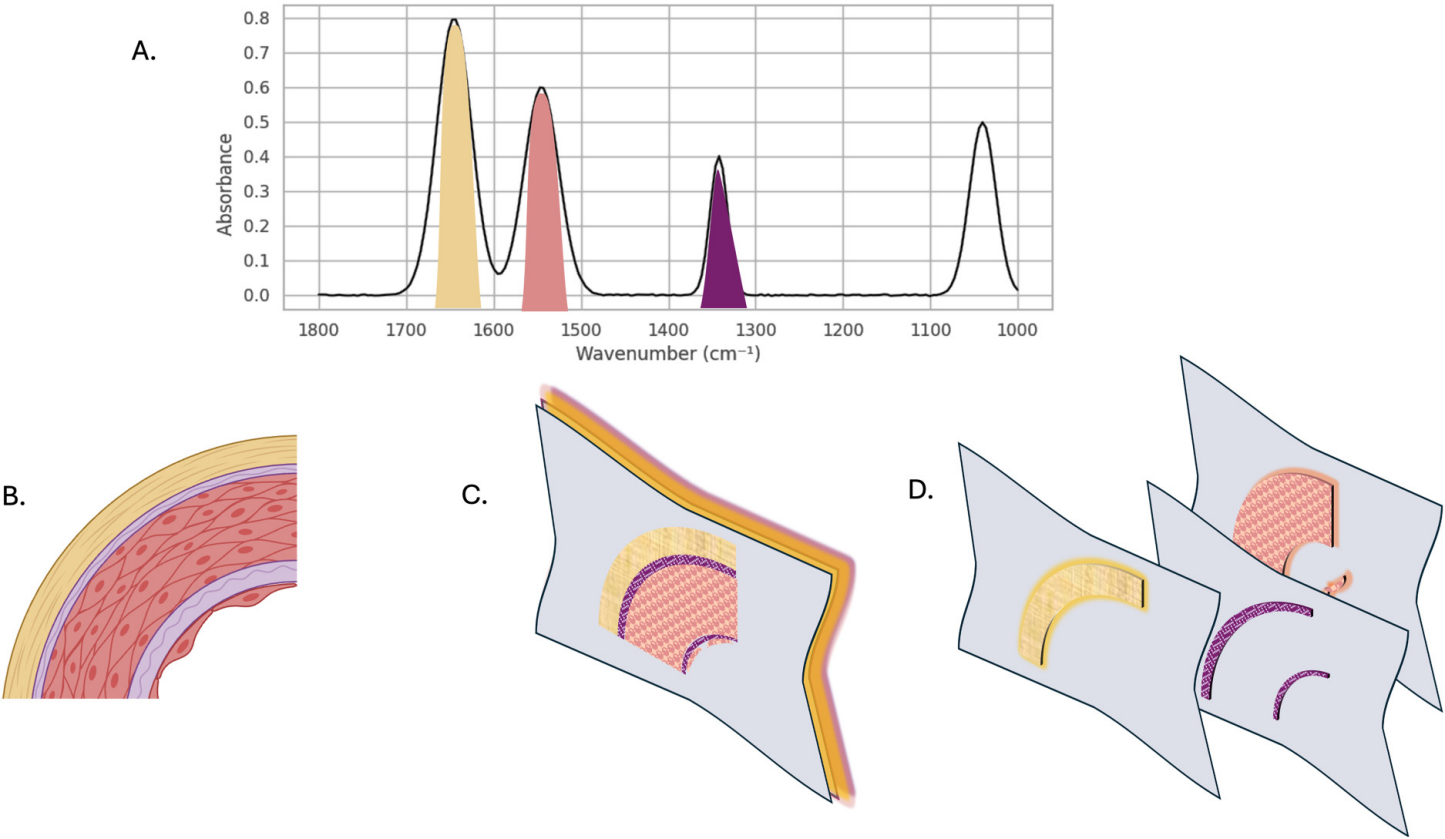
Hyperspectral versus multispectral imaging. (A) Representative spectra of a hypothetical biological sample. (B) Optical image of biological sample. (C) Hyperspectral O-PTIR image of the sample. (D) Multispectral image on various IR wavelength corresponding to absorbance in spectra from A.

In hyperspectral mode, the QCL wavelength is sequentially tuned across a defined spectral range, acquiring a full IR spectrum at each pixel (Figure 4). This produces a two-dimensional dataset (*x*, *y*) that contains both spatial and spectral information. This approach offers several important advantages, foremost among them being comprehensive molecular profiling, which allows for the identification of multiple chemical species within a single dataset. It also provides post-acquisition flexibility, meaning that spectral regions of interest can be examined later without the need to re-measure the sample, saving time and preserving sample integrity. Moreover, data-driven discovery is facilitated through unsupervised multivariate analysis techniques such as principal component analysis (PCA) and cluster analysis, which can uncover unexpected biochemical heterogeneity within the sample. These strengths make this method particularly well suited for applications such as mapping complex biological structures like tissue sections, characterizing mixed or composite materials, and detecting subtle biochemical changes associated with disease progression.

### Comparative Considerations

(i) Data Volume: Hyperspectral imaging generates much larger datasets, requiring more storage and computational resources for analysis.^82^ (ii) Acquisition Time: Single wavenumber scanning is optimal for rapid screening, while hyperspectral imaging is suited for in-depth chemical characterization.^59^ (iii) Integration with multimodal systems: Both modes can be combined with Raman or fluorescence microscopy in the same setup, enabling correlative analysis.^6^

In practice, researchers often use single wavenumber scanning as an initial screening tool to identify regions of interest, followed by hyperspectral imaging for detailed chemical analysis.^59^ This two-tiered approach maximizes both efficiency and molecular insight, making O-PTIR a highly adaptable platform for diverse scientific applications.

#### Simultaneous IR and Raman Measurements

Coherent vibrational spectroscopy (CVS) represents an innovative strategy for simultaneous infrared (IR) and Raman spectroscopy, and integrating Fourier transform infrared (FT-IR) and Fourier transform coherent anti-Stokes Raman scattering (FT-CARS) within a single interferometric platform. Using a femtosecond Ti:sapphire laser and interferometric control of the optical path difference, collinear mid-IR and near-IR pulses are generated via intra-pulse difference-frequency generation in a nonlinear GaSe crystal.^83^ The mid-IR component probes vibrational absorption, while the near-IR component simultaneously excites Raman scattering, enabling acquisition of high-resolution IR and Raman spectra from the same spatial location and time point. By leveraging ultrafast laser sources, nonlinear optics, and Fourier transform detection, CVS overcomes longstanding challenges in concurrent IR–Raman measurements, including mismatched spatial resolution, sample incompatibility, and experimental complexity.

While CVS achieved true simultaneity, its spatial resolution was limited by the diffraction of MIR light, typically in the micrometer range.^84–89^ To address this limitation, researchers turned to near-field techniques like AFM-IR,^90,91^ which detects IR absorption through the thermal expansion of a sample measured by an atomic force microscope cantilever. AFM-IR pushed spatial resolution down to ∼20  nm,^88,92,93^ but its scanning-probe nature introduced constraints such as limited field of view and complex instrumentation. Table II describes the different modalities available with advance IR instruments.

**Table II.** Evolution in simultaneous sub-micrometer IR microscopy.

Technique	Simultaneous IR and Raman	Spatial resolution	IR mechanism	Raman mechanism
CVS (FT-IR + FT-CARS)	Yes	∼1 μm	MIR via intra-pulse difference frequency generation (DFG)	Coherent Raman (CARS)
AFM-IR	No	∼20 nm	Thermal expansion detected by AFM cantilever	Not integrated
IR-PHI	No	∼300 nm	Photothermal heterodyne detection	Not integrated
O-PTIR	Yes	∼500 nm	Photothermal detection via visible laser	Spontaneous Raman via same probe

Seeking a balance between resolution and practicality, scientists developed infrared photothermal heterodyne imaging (IR-PHI),^51,94–97^ a far-field technique that uses a visible probe beam to detect IR-induced thermal changes in the sample. In IR-PHI, a mid-IR pump laser excites the sample, and the resulting local temperature change alters the reflectivity or transmission^98^ of the co-focused visible probe. This method achieves spatial resolution of ∼300–800 nm^59,63,73,78,99–102^ limited only by the optics of the visible probe^94–97^ and allows imaging over a widefield view.

The IR-PHI experiments have successfully visualized soft matter systems and biological samples like *E. coli* cells,^69^ demonstrating both sensitivity and resolution.^98,103,104–107^ Unlike AFM-IR, IR-PHI does not require contact or scanning, making it more suitable for dynamic or delicate samples.^108–112^

### Key Performance Metrics: Resolution and Sensitivity

Spatial and spectral resolution are fundamental parameters determining the performance of any vibrational spectroscopic technique. In conventional infrared (IR) microscopy, both are constrained by the physics of long-wavelength light. O-PTIR fundamentally changes these limits. This section discusses the lateral, axial, and spectral resolution of O-PTIR in detail, comparing it to conventional FT-IR and near-field methods.

#### Spatial Resolution

In O-PTIR spectroscopy, the achievable resolution is dictated not by the mid-infrared (mid-IR) wavelength, but by the shorter wavelength of the visible probe laser used to detect the photothermal effect. This is a key distinction from conventional far-field IR spectroscopy, where the resolution is fundamentally diffraction-limited by the IR wavelength according to:

(3) $$\;\Delta r_{IR}\;\approx \;\frac{0.61\;\lambda_{\mathrm{IR}}\;}{NA}$$

For a typical FT-IR system operating at 1650 cm^−1^ (λ ≈ 6.06  µm) with a high NA of 0.6:

(4) $$\Delta r_{IR}\;\approx \;\frac{0.61\;\times 6.06\;}{0.6}\approx 6.15\;\mu m$$

This resolution is far too coarse for subcellular imaging. In O-PTIR, however, the probe beam is usually in the visible range (e.g., 532  nm or 785  nm), so the diffraction-limited lateral resolution for λ = 532  nm and NA = 0.8:

(5) $$\Delta r_{OPTIR}\;\approx \;\frac{0.61\;\times 0.532\;}{0.8}\approx 0.405\;\mu m$$

Thus, O-PTIR can achieve sub-micrometer spatial resolution, roughly 10–15× better than conventional IR microscopy. To achieve this high resolution, there are considerations like, the IR beam must fully cover the probe beam's focal spot for optimal resolution. In reflection mode, resolutions of 0.4–0.5  µm are typical, with best-reported values around 0.35  µm under optimal alignment. Prandini et al. have used the O-PTIR with spatial resolution of 0.416  µm for analyzing the crystals.^63^ Although superior to far-field FT-IR, O-PTIR remains diffraction-limited by the visible probe wavelength, so it does not reach the nanoscale capabilities of AFM-IR (20–50  nm).^113,114^

#### Axial Resolution

O-PTIR achieves significantly improved axial resolution because it is defined by the depth of focus of the visible probe beam rather than the IR excitation wavelength. The theoretical axial resolution can be expressed as^1^:

$$\Delta Z_{OPTIR\;}\approx \;\frac{2n\;\lambda_{Vis}}{NA^{2}}$$

where *n* is the refractive index of the medium, λ_vis_ is the probe wavelength, and NA is the numerical aperture of the objective lens.

For a typical O-PTIR configuration using a 532  nm probe, NA = 0.8, and n ≈ 1 (air objective), the calculated axial resolution is:

(7) $$\Delta Z_{OPTIR\;}\approx \;\frac{2\;\times 1\times 0.532}{0.8^{2}}\;\approx 1.66\;\mu m$$

The researchers reported the axial resolution of 1.7 μm, which is in agreement with the theoretical values.^63^ This sub 2  µm depth resolution enables selective interrogation of thin sections or specific focal planes within thicker samples, representing a 5–10× improvement over conventional IR techniques and facilitating depth-resolved chemical imaging.

### Spectral Resolution

Spectral resolution in O-PTIR is decoupled from spatial resolution and is primarily determined by the linewidth of the IR excitation source and the chosen frequency step size. Table III compares the resolution across different IR instruments. In most modern O-PTIR instruments, the IR source is a quantum cascade laser (QCL), which offers narrow linewidth operation and tunability. QCL based O-PTIR typically achieves spectral resolutions of 1–2 cm^−1^, with specialized configurations reaching 0.5 cm^−1^. Broadband FT-IR-based O-PTIR, systems can match conventional FT-IR performance, offering resolutions in the range of 0.5–4 cm^−1^, depending on interferometer settings and optical path length. This flexibility allows O-PTIR to be optimized for either high spectral fidelity (narrow linewidth, fine frequency steps) or faster acquisition (coarser resolution), without compromising its superior spatial resolution.

**Table III.** Comparison in resolution of FT-IR versus O-PTIR versus AFM-IR.

Parameter	Conventional FT-IR microscopy	O-PTIR	AFM-IR/sSNOM
Spatial resolution (*x*,*y*-plane feature discrimination)	3–15 µm (set by IR diffraction limit)	∼0.4–0.5 µm typical; ∼0.35 µm best-reported^6,59,99,102^	∼20–50 nm typical; can reach <10 nm in optimized s-SNOM. Resolution set by AFM tip radius, not diffraction
Axial (depth) resolution (*z*-plane discrimination)	~1–3 µm (IR depth of focus). Multiple layers contribute to signal; poor depth selectivity	∼1–3 µm typical. Depth set by visible probe's depth of focus^71^	∼20–50 nm depth sensitivity
Spectral resolution (Frequency discrimination)	0.5–16 cm^−1^ depending on interferometer settings	2–8 cm^−1^ typical for narrow-line QCL sources. Step-scan or tunable laser control^1,71^	0.5–8 cm^−1^ typical; limited by laser source linewidth, not spatial resolution
Resolution-limiting Factors	IR wavelength and NA of objective	Visible probe wavelength and NA^1^	AFM tip size, tip–sample coupling efficiency, and mechanical stability
Typical applications by resolution	Tissue mapping, polymer blends, bulk chemical ID	Subcellular mapping (organelles, bacteria), microplastics, fine compositional gradients	Nanodomain analysis in biomembranes, protein aggregates, polymer nanophases
Relative improvement over FT-IR	–	∼10–15× better lateral spatial resolution, ∼5–10× better axial resolution	>100× better lateral and axial resolution

#### Sensitivity of O-PTIR

Sensitivity in O-PTIR is determined by how small a temperature change can be detected via the probe beam. The photothermal effect produces a refractive index change (Δ*n*) and/or surface displacement proportional to the IR absorption and thermal properties of the sample:

(8) $$\Delta T\;\propto \;\frac{\alpha I_{IR}}{\rho C_{p}}$$

where α is absorption coefficient, I_IR_​ is incident IR intensity, ρ is density of the material, and Cp is heat capacity.

Modern O-PTIR instruments can detect refractive index changes corresponding to temperature rises on the order of 10^–4^/°C which translates to the detection of sub-monomolecular surface coverages in favorable conditions.^1^ This sensitivity is often limited by thermal conductivity, laser power stability, detector noise, and environmental vibrations. Compared to conventional IR, O-PTIR can achieve similar or better sensitivity on small features because the visible probe efficiently collects scattered/perturbed light from micrometer or sub-micrometer spots whereas in conventional IR, the diffraction-limited spot size reduces the local photon density and signal strength.

## Comparison with Other Spectroscopic Techniques

### O-PTIR Versus Traditional FT-IR and Raman Spectroscopy

For decades, FT-IR and Raman spectroscopy have been central to molecular characterization in life sciences and materials research, offering complementary insights into molecular vibrations. However, both techniques face intrinsic limitations at the microscale, particularly for biological samples. FT-IR is constrained by the mid-IR diffraction limit, restricting spatial resolution to several micrometers and limiting its ability to resolve subcellular features, while also requiring careful sample preparation and being susceptible to scattering artifacts. Raman spectroscopy provides sub-micrometer spatial resolution but is often hindered by weak intrinsic signals and strong fluorescence backgrounds in biological specimens, reducing sensitivity to low-abundance species. O-PTIR emerged to address these challenges by detecting IR-induced photothermal responses with a visible probe laser, thereby decoupling spatial resolution from IR wavelength and enabling imaging below 500  nm. Moreover, the commercial O-PTIR allows simultaneous IR and Raman acquisition from the same region, delivering complementary vibrational information within a single experiment and significantly advancing microscale chemical imaging. Table IV compare the different IR technologies available in market.

**Table IV.** Comparison between different IR based technologies available in the market.

Feature	FT-IR	QCL-IR	AFM-IR	O-PTIR
IR source	Thermal globar or synchrotron	Quantum cascade laser (QCL)	QCL or optical parametric oscillator (OPO)	QCL or OPO (pump) + visible laser (probe)
Detection principle	Measures residual IR radiation	Measures residual IR radiation	Measures photothermal IR effect via cantilever oscillation^116^	Measures photothermal IR effect via refractive index change
Spectral range	Broad (4000–400 cm^−1^) or broader with synchrotron (8000-200 cm^−1^)	Tunable; now covers full fingerprint region	Depends on laser; similar to O-PTIR	Depends on laser: ∼1800–800 cm^−1^; up to 3600–800 cm^−1^ with dual lasers
Fourier transform needed?	Yes	No^117^	No	No
Spatial resolution	∼3–15 µm	∼10–20 µm	∼20 nm^118^	Sub-micrometer^119^
Modes of operation	Transmission, reflection, ATR	Same as FT-IR	Single-point, single-frequency	Single-point, single-frequency, hyperspectral
Imaging speed	Moderate to slow (depends on detector)	Fast, especially with focal plane array (FPA)^54,70^	Very slow (point-by-point)	Slow (single-element detector)
Signal-to-noise ratio (SNR)	Moderate	High	High	High
Artifacts	Susceptible to scatter artifacts	Laser coherence artifacts possible^56^	Minimal	Immune to dispersive scatter artifacts^6^
Sample contact	ATR requires contact	ATR requires contact	Contact via AFM-tip	Non-contact
Sample size suitability	µm scale^57^	µm scale	nm scale	Sub-µm scale
Raman integration	No	No	No	Yes (Simultaneous IR + Raman)
Best use case	General analysis, historical standard	Fast, targeted	Nanoscale and surface structure analysis	High-resolution, artifact-free imaging + Raman

The advantages of O-PTIR are not merely theoretical. In a 2024 comparative study, Richardson et al.,^115^ evaluated both FT-IR and O-PTIR for their ability to distinguish closely related bacterial species *Staphylococcus capitis*, *Staphylococcus epidermidis*, and *Micrococcus luteus*. Using a Bruker FT-IR system and O-PTIR, they found that even small amounts of biomass yielded highly discriminative spectra using O-PTIR, on par with FT-IR. Principal component discriminant function analysis (PC-DFA) successfully separated the bacterial species and even distinguished between sub-species, with validation models confirming classification robustness. These results underscore O-PTIR's potential for single-cell resolution bacterial analysis, paving the way for high-precision microbiological diagnostics and phenotyping.

Beyond microbiology, the capabilities of O-PTIR have been benchmarked across a wider spectrum of vibrational techniques. In a comprehensive work, Farnesi et al., in 2024,^72^ compared O-PTIR with Raman, surface-enhanced Raman spectroscopy (SERS), hyperspectral stimulated Raman scattering (SRS), and broadband coherent anti-Stokes Raman scattering (CARS). Across criteria such as background suppression, acquisition speed, and quantitative potential, O-PTIR consistently outperformed or matched the leading techniques, all while maintaining a label-free, non-contact measurement approach.

However, as with any emerging technology, O-PTIR is not without its challenges. While its technical capabilities are impressive, the method is still maturing. Instrument availability, cost, and limited standardization across laboratories are current barriers to widespread adoption. Yet, these are common growing pains for any disruptive analytical technology in its early phase, many of which fade as the field embraces its utility. O-PTIR stands not as a replacement but as an evolution of vibrational spectroscopy. By uniting the chemical richness of infrared absorption with the spatial finesse of optical detection, it addresses many of the limitations of FT-IR and Raman spectroscopy. As more researchers begin to leverage its full potential, O-PTIR is poised to become a central tool in high-resolution, chemically specific imaging across biological and biomedical sciences.

### Complementary Nature of IR and Raman Data

The complementary nature of IR and Raman spectroscopy is well established in the field of molecular analysis. IR spectroscopy is sensitive for detecting polar functional groups, such as phospholipids, proteins, and nucleic acids. Raman spectroscopy, in contrast, is sensitive for detecting non-polar bonds and symmetric molecular structures.

A compelling demonstration of the complementary power of O-PTIR and Raman data is seen in recent work applying simultaneous SERS and O-PTIR imaging to characterize subpopulations within SW620 colon cancer cells.^58^ The cells were sorted based on integrin α5β1 activity using c-RGDfC-functionalized magnetic nanoparticles, and the two subgroups were spectroscopically analyzed. SERS successfully tracked receptor–ligand binding, while hyperspectral O-PTIR mapping, analyzed via PLS-DA, revealed biochemical differences consistent with distinct cell cycle phases. This study highlights how the integration of infrared and Raman modalities allows discrimination of functional subpopulations that might otherwise go undetected by either method alone demonstrating the synergistic value of multimodal vibrational imaging in cellular research.

By integrating IR and Raman spectroscopy, the commercially available O-PTIR provides a comprehensive view of the sample's chemical composition and structure. Simultaneous acquisition of IR and Raman data eliminates the need for sample repositioning or re-alignment, reducing experimental variability and improving data correlation. This multimodal approach is particularly valuable in biological research, where complex samples often exhibit both polar and non-polar molecular features. For example, in cellular and subcellular analysis, IR spectroscopy can identify polar biomolecules such as lipids and proteins, while Raman spectroscopy can detect non-polar molecules like cholesterol and carotenoids.^2^ The ability to obtain complementary information from a single sample region enhances the depth and breadth of chemical analysis, providing more accurate and insightful results.

### Significance of IR and Raman Spectroscopy in Biological Research

Infrared and Raman spectroscopy are foundational tools in the field of molecular biology and biomedical research. Both techniques provide vibrational spectra that serve as molecular fingerprints, enabling the identification and characterization of a wide range of biomolecules, including proteins, lipids, nucleic acids, and carbohydrates. Table V shows a comparison between Raman and Infrared spectroscopy. In biological research, these techniques are used to analyze the biochemical composition of cells and tissues, monitor metabolic changes and disease progression, identify biomarkers for diagnostics and therapeutics, study drug–cell interactions and pharmacokinetics and many more.

**Table V.** Comparison between Raman and infrared spectroscopy.

Aspect	Raman spectroscopy	Infrared spectroscopy
Principle	Measures inelastic scattering of photons (Raman effect); detects changes in polarizability of molecules	Measures absorption of IR radiation; detects changes in dipole moment of bonds
Type of energy measured	Raman shift (energy difference between incident and scattered photons)	IR absorption at specific vibrational frequencies
Radiation source	Monochromatic laser (visible or NIR)	Broad band IR source (globar) or synchrotron
Detector	CCD detector with holographic grating	Pyroelectric (DTGS) or photonic (MCT) detector with interferometer
Molecular information provided	Intra- and intermolecular vibration, crystal lattice modes, polymorphism	Intramolecular vibrations, fingerprint region of molecular bonds
Function group sensitivity	Sensitive to non-polar bonds and symmetric vibrations (e.g., C═C, S–S, C–C, N═N)	Sensitive to polar bonds and functional groups with dipole moment (e.g., N═O, O–H, C═O)
Water interference	Minimal (O–H are not Raman-active), suitable for aqueous samples	Strong water absorption in mid-IR, problematic in transmission but mitigated by ATR FT-IR
Fluorescence interference	Major limitation: fluorescence can overwhelm Raman signal	Not affected by fluorescence
Sample preparation	Minimal; works with solids, liquid, gels, or films	Minimal; ATR FT-IR eliminates need for complex preparation
Sample interaction	Focused laser beam via sapphire lens, immersion, non-contact, or flow cell	ATR crystal (diamond, Silicon, ZnSe) immersion or flow cell
Fiber optic probe compatibility	Long range (meters to kilometer); silica based	Limited range (<4 m); requires chalcogenide or AgX fibres
Quantification capability	Semi-quantitative; signal proportional to number of bonds	Strong IR bands; suitable for univariate/multivariate quantification
Use in reaction monitoring	Excellent for non-polar species, polymorphs, crystal in solution, aqueous systems	Excellent for polar species, functional group analysis, intermediates, and kinetic profiling
Common applications	Crystallization studies, polymorph analysis, reaction monitoring in aqueous/harsh conditions, catalyst research	Reaction kinetics, structure elucidation, catalyst monitoring, polymer side-chain analysis, fingerprinting
Limitation	Fluorescence, not all molecules are Raman active	Water absorption, saturation for strong absorbers in transmission mode

### Advantages of Submicrometer Resolution in Biological Applications

O-PTIR spectroscopy overcomes the resolution barriers providing a unique window into the capturing chemical heterogeneity and molecular organization in complex biological systems with remarkable clarity.

**Figure 5.**
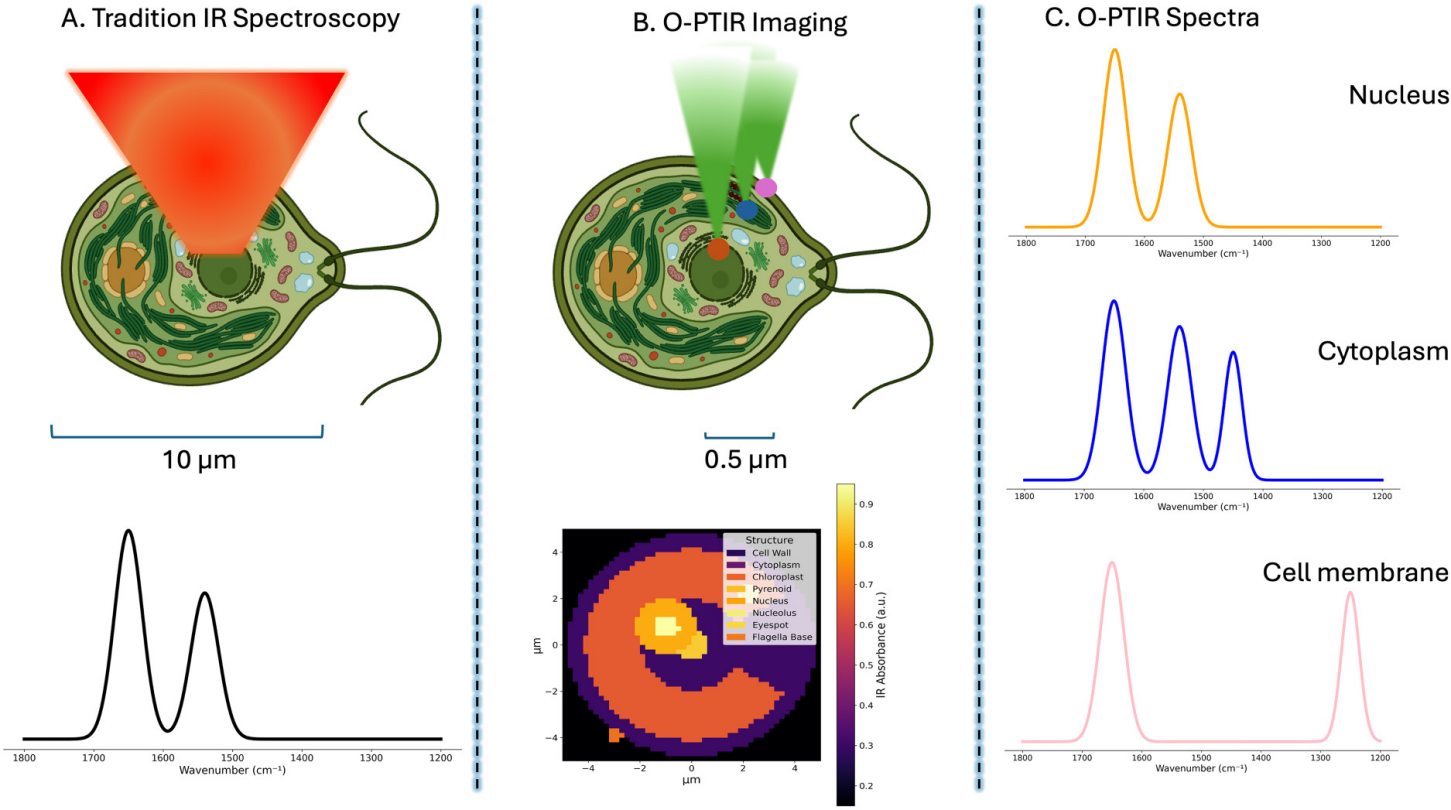
Comparison between traditional IR versus O-PTIR in analyzing biological cells.

This enhanced spatial resolution proves invaluable in numerous biomedical contexts. In cancer diagnostics, for example, O-PTIR enables the identification of molecular alterations at the subcellular level,^59^ such as changes in protein secondary structures or lipid distributions, which can reflect disease progression or treatment response. In microbial research, it allows for the classification of bacterial species^3^ based on subtle differences in their biochemical signatures, even at the single-cell level. These applications highlight the power of sub-micrometer resolution to reveal biologically meaningful patterns that remain hidden in conventional spectroscopy. Figure 5 represent the comparison between traditional IR versus O-PTIR in analyzing biological cells, where the figure represents mock spectra of the sub cellular components.

The benefits of high-resolution spectroscopy become even more pronounced when investigating heterogeneous tissues, such as bone. A compelling example comes from bone matrix analysis, where assessing the ratio between organic matrix and mineral content is critical for understanding structural maturity.^120^ While FT-IR and Raman often average over regions several micrometers wide, techniques like AFM-IR have shown that matrix-to-mineral domain sizes can range from 50–700  nm, with sharp compositional transitions occurring within just 100–200  nm. These details are undetectable when using coarse spatial averaging typical of traditional methods. O-PTIR further supports these findings by revealing composite mineralization patterns, such as 20–40 μm extended mineral domains embedded with 1–5 μm micro-heterogeneities. Notably, younger bone tissue exhibits more variation in mineral content due to the irregular infilling of a collagen framework patterns that correlate well with mechanical testing results like nanoindentation. In this way, the sub-micrometer imaging capability of O-PTIR enables a multimodal, multi-scale understanding of tissue development and pathology.

Beyond structural insight, O-PTIR's fine resolution also enhances tissue classification in biomedical imaging. Conventional FT-IR imaging captures biochemical spectra averaged over ∼5 μm pixels, masking critical subcellular variations. By contrast, O-PTIR imaging at ∼0.5 μm resolution can discern biochemical heterogeneity across cell nuclei, cytoplasm, and organelles. However, this increase in resolution can introduce greater spectral variability within tissue classes, potentially complicating automated classification tasks.

To address this, researchers have integrated O-PTIR with deep learning techniques, such as convolutional neural networks (CNNs), to improve classification performance despite the added spectral complexity. In a recent study analyzing ovarian cancer tissues from 78 patients, a CNN model trained on a reduced set of mid-IR bands acquired using O-PTIR achieved an area under the receiver operating characteristic curve (AUC) of 0.98, outperforming traditional random forest models (AUC = 0.58).^80^ This success illustrates that even with fewer spectral features, enhanced spatial resolution can drive improved diagnostic accuracy. Moreover, such high-resolution imaging allows for pixel-level segmentation of tissue components, enabling the extraction of quantitative biomarkers such as the stromal ratio (SR) and epithelium ratio (ER). These metrics have shown strong correlation with cancer grade and progression, underscoring the clinical potential of combining O-PTIR's spatial precision with robust analytical frameworks.

The sub-micrometer resolution capability of O-PTIR extends far beyond image clarity, it enables molecular-level insight, improved diagnostic precision, and deeper mechanistic understanding of biological systems. As biological research increasingly demands high-resolution, label-free, and chemically specific techniques, O-PTIR stands out as a transformative tool for the next generation of cellular and tissue analysis.

## Applications of O-PTIR in Biological Research

### Cellular and Subcellular Analysis

#### Detection of Biomolecules (Lipids, Proteins, Nucleic Acids)

O-PTIR allows for high-resolution, label-free imaging of lipids, proteins, nucleic acids, and other biomolecules by fusing the spatial accuracy of visible light detection with the molecular specificity of infrared (IR) absorption. This method offers comprehensive chemical maps that show the concentration and distribution of biomolecules inside cells, providing information about the structure and operation of cells at a subcellular level.

The creation of fluorescence-guided optical photothermal infrared spectroscopy (FL-O-PTIR), which combines fluorescence imaging and mid-infrared photothermal spectroscopy to target cells, organelles, and molecules within complex biological samples, is an exciting development in this field.

By employing fluorescence to direct spectral acquisition, FL-O-PTIR gets over difficulties with localizing data collection points. As an example, Prater et al, have successfully located and analyzed amyloid proteins and related microglia cells directly in brain tissue, this method has made it possible to investigate β-sheet architectures at the subcellular scale.^73^ Furthermore, FL-O-PTIR has been used to detect amyloid aggregation and susceptible neuronal areas with low cell stress in cultured primary neurons treated with amyloid-beta peptides. FL-O-PTIR offers a potent platform for high-resolution biochemical mapping in biological tissues and cells by combining fluorescence and infrared imaging. It has potential uses in the investigation of protein misfolding disorders and other cellular processes.

Collagen, a crucial structural biomolecule,^121^ has been successfully detected and characterized using fluorescence detection photothermal infrared (FL-PTIR). Collagen presents difficulties for traditional infrared or fluorescence-based methods because of its inherent autofluorescence, which is caused by tyrosine residues^122^ and/or crosslinks.^123^ In this regard, Prater et al.^2^ showed that O-PTIR offers comprehensive chemical information and permits label-free collagen identification. They obtained autofluorescence and infrared chemical pictures of collagen samples using FL-PTIR. They also demonstrated that spectral characteristics, such as amide I and II bands, changed with fibril orientation, demonstrating the technique's sensitivity to both structural organization and molecular presence.

#### Monitoring Metabolic Activities and Biochemical Changes

O-PTIR is a powerful tool for monitoring metabolic activities and biochemical changes within cells. By detecting the photothermal response of biomolecules to IR radiation, O-PTIR can track dynamic processes such as protein folding, lipid metabolism, and nucleic acid interactions. This capability is particularly valuable for studying cellular responses to external stimuli, drug treatments, and disease progression. O-PTIR's high sensitivity and spatial resolution enable the detection of subtle biochemical changes, providing a deeper understanding of cellular metabolism and function.

Recent developments have expanded O-PTIR applications into single-cell metabolic imaging with unprecedented chemical specificity and spatial resolution.^123^ A metabolic imaging platform utilizing azide-labelled palmitic acid (PA) demonstrated direct imaging of lipid metabolism in fixed cells, with potential for extension to live-cell studies. Compared to fluorescence-based optical metabolic imaging (OMI) and vibrational techniques such as spontaneous Raman and stimulated Raman scattering (SRS), O-PTIR offers several unique advantages: negligible fluorescence interference, the ability to switch between single-color and full-spectrum modes, and reduced instrumentation complexity by eliminating ultrafast laser requirements.

Notably, this study highlighted key trade-offs between spatial and spectral resolution, throughput, and image segmentation accuracy. Despite limited wavenumber sampling, deep learning analysis of O-PTIR data (using azide labels) revealed robust classification of lipid metabolic activity across tens to hundreds of cells. With further throughput enhancements such as widefield O-PTIR, sparse spectral sampling, and denoising algorithms, this approach could scale to larger cell populations for statistically robust studies.

The platform's flexibility extends to other metabolic targets, including cholesterol, which plays a crucial role in neurodegenerative diseases.^123^ Additionally, the estimated thermal impact of the O-PTIR signal (∼2.9  K) remains within safe bounds for live-cell imaging, further supporting its suitability for longitudinal studies. Applications to granulin (GRN)-deficient human induced pluripotent stem cell (iPSC)-derived models revealed elevated lipid metabolism, with future extensions planned to organoids and animal models.^123^ Beyond neuroscience, this high-resolution metabolic platform may also enable single-cell analysis in microbiology for energy optimization and biofuel production.

O-PTIR has proven highly effective in monitoring lipid metabolic activity at the single-cell level. One notable application is the tracking of de novo lipogenesis (DNL) in both fixed and live adipocytes using azide-labelled metabolic probes.^124^ In this study, O-PTIR enabled spatially resolved quantification of lipid synthesis rates within and between individual cells. Hyperspectral imaging of fixed cells provided comprehensive molecular insight, including detailed lipid and protein signatures, while single-frequency live-cell imaging preserved cell morphology and subcellular lipid localization. Although each modality offers distinct advantages, together they deliver a more complete picture of metabolic activity in adipose tissue. This dual-mode capability highlights O-PTIR's strength in resolving dynamic biochemical processes relevant to both healthy and disease states.

The subcellular capabilities of O-PTIR can be significantly enhanced when combined with complementary modalities like SERS. In a study investigating SW620 colon cancer cells,^58^ researchers used a peptide-functionalized nanoparticle sorting method to distinguish subpopulations based on integrin α5β1 activity. Simultaneous O-PTIR and SERS measurements revealed molecular differences tied to receptor binding and cell cycle progression (G1 to S phase). With >80% classification accuracy using PLS-DA on O-PTIR spectra, this approach exemplifies how spectroscopic multimodality enables detection of subtle functional heterogeneity within cell lines. This has strong implications for drug testing, cancer progression models, and cell-based screening.^125^

### Microbial Studies

#### Identification and Classification of Microbial Species

O-PTIR has emerged as a valuable technique for the identification and classification of microbial species. By analyzing the IR absorption spectra and Raman spectra of microbial cells, O-PTIR can differentiate between species based on their unique molecular fingerprints. This capability is essential for studying microbial diversity, ecology, and evolution. O-PTIR's high-resolution imaging allows for the detection of individual microbial cells and colonies, facilitating the identification of rare or novel species.

Florescence PTIR has been employed for the label-free detection and chemical imaging of photosynthetic microorganisms, leveraging their strong autofluorescence signals, particularly from chlorophyll^2^ as well as their rich biochemical content. In recent work, diatoms and green microalgae were investigated using FL-PTIR, enabling high-resolution mapping of protein and silica content in individual microbial cells.^2^ For diatoms, IR absorption images and spectral data were collected with sub-micrometer spatial resolution, revealing variations in silica polymerization (Q3/Q4 ratios) and protein distribution. The analysis also demonstrated that pore structures within diatom frustules exhibit distinct chemical composition, not resolvable using conventional FT-IR microscopy.

Similarly, auto-fluorescent green microalgae (putatively *Monoraphidium* spp.) were studied in both dried and live states.^2^ Hyperspectral FL-PTIR imaging enabled acquisition of spatially resolved IR spectra from single cells and, notably, dynamic measurements in aqueous environments. This approach highlights the potential of FL-PTIR for non-destructive, high-speed analysis of live microorganisms, offering both structural and functional insights without the need for external labels.

#### Understanding Microbial Interactions and Metabolism

O-PTIR provides insights into microbial interactions and metabolism by revealing the chemical composition and spatial distribution of biomolecules within microbial communities. This technique can track metabolic exchanges between microbial cells, monitor the production of metabolites, and study the effects of environmental changes on microbial activity. O-PTIR's ability to perform label-free, non-destructive imaging makes it ideal for studying live microbial cultures and their interactions with other organisms.

In a recent study, Lima et al.^3^ demonstrated the use of O-PTIR spectroscopy to monitor bacterial cell metabolism by tracking the incorporation of stable carbon and nitrogen isotopes (^13^C and ^15^N). The researchers conducted simultaneous IR and Raman measurements and compared the results with traditional techniques such as FT-IR spectroscopy, highlighting the enhanced sensitivity and spatial resolution of O-PTIR. This study represents a notable example of using IR spectroscopy to detect isotopic shifts in biological systems. A similar study was conducted by Shuster et al.,^8^ demonstrating the incorporation of ^13^C-labeled glucose in *E. coli*, along with azide labelling of cellular components. The researchers also performed live-cell imaging in both water and buffer solutions, highlighting the capability of monitoring metabolic activity in real-time under physiologically relevant conditions.

A particularly innovative advancement is the integration of O-PTIR with fluorescence in situ hybridization (FISH), a technique known as O-PTIR-FISH (see FISH-O-PTIR: Integrating Fluorescence In Situ Hybridization with O-PTIR for Single-Cell Metabolic Profiling section below). This platform enables simultaneous species identification and metabolic analysis within complex microbial communities. For example, bacteria cultured with ^13^C-glucose exhibit isotope-induced red shifts in O-PTIR spectra, which correlate with metabolic activity. When combined with rRNA-tagged FISH probes for cell identity, this method facilitates single-cell resolution analysis in mixed populations such as the human gut microbiome.^126^ Such an approach is invaluable for dissecting community structure–function relationships and for investigating microbial roles in health and disease.

### Tissue and Disease Diagnostics

In cancer diagnostics, O-PTIR has played a crucial role in revealing the nanoscale composition of pathological mineral deposits. Specifically, studies of breast microcalcifications using O-PTIR alongside AFM-IR have uncovered chemical heterogeneity and the presence of mineral phases beyond the traditional classification of calcium oxalate dihydrate and calcium phosphate apatite.^127^ These sub-micrometric insights offer promising avenues for improving the pathological interpretation of suspicious calcifications detected by mammography, especially in early biomineralization stages. Such capabilities suggest O-PTIR's potential utility in longitudinal tracking of disease progression and therapeutic response.

Beyond oncology, O-PTIR addresses challenges in nephrology and other fields where crystalline pathologies are prevalent.^128^ While conventional FT-IR remains the gold standard for bulk kidney stone analysis, its limited spatial resolution hampers the characterization of microcalcifications smaller than 10 μm. O-PTIR overcomes this limitation by enabling spectral acquisition at sub-micrometer resolution without the need for special sample preparation. This has facilitated precise discrimination among calcium oxalate monohydrate, calcium oxalate dihydrate, calcium phosphate apatite, and magnesium ammonium phosphate hexahydrate, as well as detailed analyses of pathological calcifications associated with conditions such as hyperoxaluria, APRT deficiency, and Randall's plaque. The technique has also been extended to breast calcifications, though differentiating closely related calcium phosphate phases such as amorphous carbonated calcium phosphate and whitlockite remains challenging, highlighting current limitations alongside O-PTIR's advantages.

In the study of musculoskeletal health, O-PTIR, often combined with AFM-IR and Raman spectroscopy, has provided new insights into bone tissue aging and microstructural heterogeneity.^120^ By mapping matrix-to-mineral ratios at nano- to microscale resolution, these studies revealed abrupt mineralization transitions occurring within 100–200  nm and identified banded structures and domain sizes ranging from approximately 50  nm to several micrometers. Notably, younger bone tissue showed greater compositional variability than mature bone, findings that not only complement results from bulk FT-IR and Raman spectroscopy but also uncover nanoscale heterogeneity missed by conventional methods. These observations have important implications for understanding bone development, remodeling, and fragility.

O-PTIR has also been adapted to detect and chemically identify exogenous contaminants such as microplastics in human tissues.^129^ In a pioneering workflow applied to formalin-fixed paraffin-embedded colon tissue sections, O-PTIR successfully localized polyethylene, polystyrene, and polyethylene terephthalate particles and correlated their presence with histopathological signs of inflammation in adjacent tissue regions. This non-destructive approach integrates seamlessly with routine pathology protocols and opens new possibilities for exploring environmental health impacts in clinical contexts one of such advances is, detection of microplastics in the tissue samples.^130^

Optical photothermal infrared spectroscopy bridges the gap between high-resolution imaging and molecular specificity in tissue diagnostics. Its ability to resolve nanoscale chemical heterogeneity in mineralized deposits, map immune cell distributions, monitor biochemical transformations, characterize age-related bone changes, and detect environmental contaminants underscores its versatility across biomedical and clinical research domains. The non-destructive nature of the technique and its compatibility with standard histopathology enhance its translational potential. Nonetheless, challenges such as spectral overlap between closely related mineral phases and matrix effects from surrounding biological components remain. Ongoing methodological improvements and integration with complementary techniques like AFM-IR, Raman spectroscopy, and conventional histology are anticipated to expand O-PTIR's role in mechanistic studies and clinical diagnostics alike.

### Drug Delivery and Pharmaceutical Research

In pharmaceutical manufacturing, one of the most critical aspects of quality control is verifying the uniform distribution of active pharmaceutical ingredients (APIs) within solid dosage forms like tablets. Uneven API distribution can directly impact a drug's efficacy, safety, and regulatory compliance, and is a common cause of product recalls. Achieving high spatial resolution while maintaining chemical specificity is essential to detect inhomogeneities and ensure content uniformity.^131^

Conventional infrared (IR) microscopy, while chemically informative is often too coarse to resolve fine particulate distributions.^132–134^ Raman spectroscopy suffers from weak signals and longer acquisition times, and coherent Raman techniques, although powerful, are costly and not yet practical for widespread industrial adoption.

Fluorescence-based methods have been used before to study pharmaceutical materials because many small-molecule drugs naturally contain aromatic groups that glow (fluoresce) under UV light. For example, Chen et al.^135^ and colleagues developed a UV–fluorescence tool to detect a drug called amiloride in tablets and blood samples. Toth et al.^136^ and his team also showed that UV fluorescence methods can be quite good at detecting drugs.

However, using fluorescence alone can be difficult because many common inactive ingredients in tablets (like TiO_2_ or HPMC) also glow under UV light, making it harder to tell the drug apart from everything else. Tiny impurities left over from making the drug can also add unwanted background signals. On top of that, other things like surface defects,^137^ amine–oxygen exciplex formation,^138^ and proteinaceous airborne particulates^139^ can also create confusing signals.

Another problem is that the glowing signals from different sources often overlap and are hard to separate clearly. And because UV light tends to produce short-lived signals, it limits how much useful information we can get from their timing. Therefore, using methods that focus more directly on the molecular structure of the drug like infrared spectroscopy while still taking advantage of the natural glow (autofluorescence) of certain ingredients help to improve visibility and contrast.

Photothermal IR methods overcome the resolution barrier by detecting local thermal expansion caused by IR absorption and when aided with fluorescence it becomes autofluorescence-detected photothermal IR (AF-PTIR) which introduces a significant advancement by leveraging the natural UV autofluorescence of many APIs and proteins.^4^ By using fluorescence as a sensitive probe to detect IR-induced thermal changes, AF-PTIR offers several advantages like label-free selectivity, high spatial resolution, improved sensitivity and contrast along with multimodal compatibility. Razumtcev et al.^4^ have used the same concept to map the indomethacin (API) particles with high sensitivity using AF-PTIR in powdered mixture of common excipients like titania powder. AF-PTIR has the potential to aid in assessing content uniformity in pharmaceutical tablets production by rapidly visualizing the spatial distribution of active pharmaceutical ingredients within final dosage forms.^4^ It is also interesting to consider possible future applications of AF-PTIR for analysis of therapeutic macromolecular formulations of protein-based biologics. Previous demonstration of UV–fluorescence in proteins^140^ along with preliminary proof-of-concept AF-PTIR measurements of tryptophan microcrystals and lyophilized protein particles^61^ suggests potential compatibility with biological formulations analysis.

## Future Directions and Emerging Trends

### Advances in Live-Cell O-PTIR Measurements

**Figure 6.**
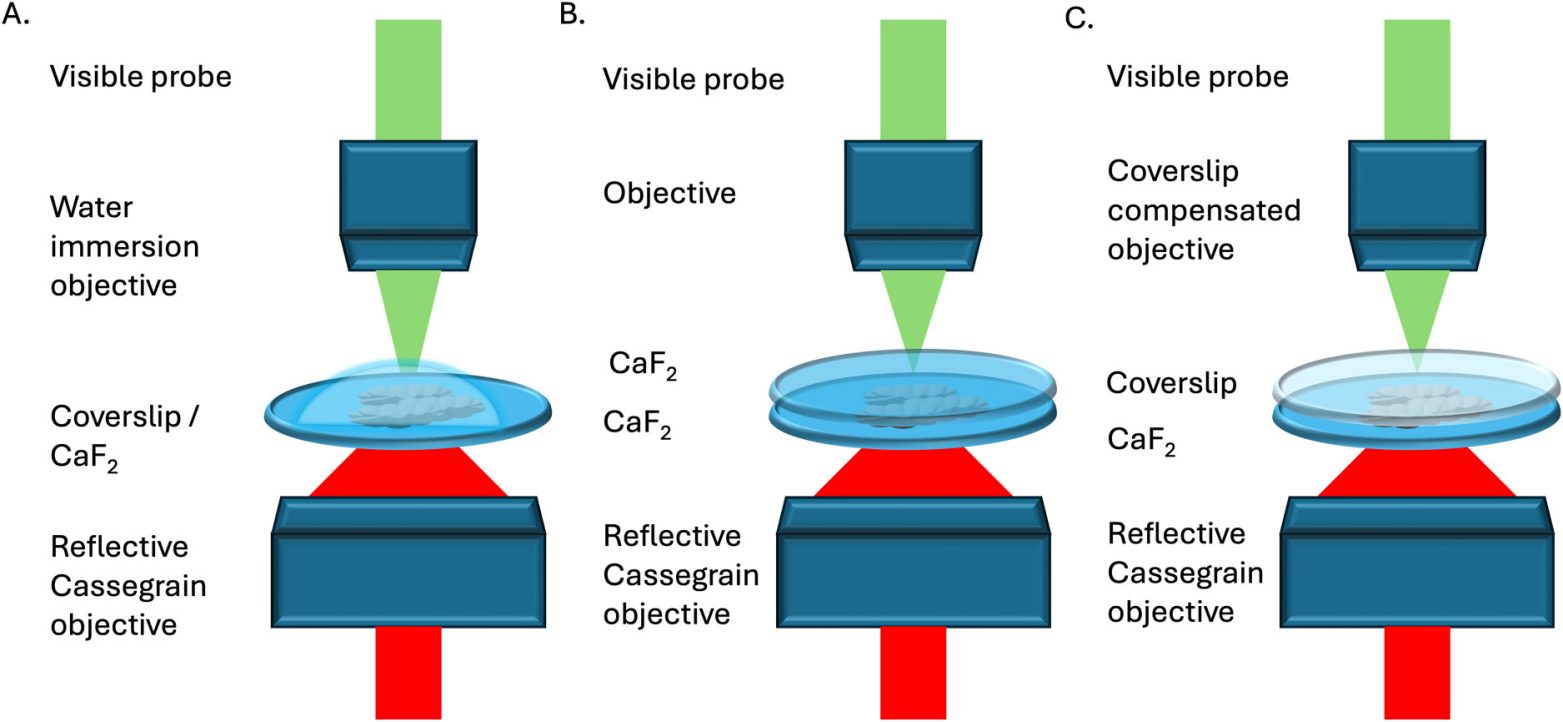
Different modes associated with live cell measurements in counter propagation mode in O-PTIR.

Live-cell imaging has advanced significantly with the integration of O-PTIR (Figure 6), which enables high-resolution, label-free chemical imaging of dynamic cellular processes that are difficult to probe using conventional IR spectroscopy. There are various setups that can be adapted to perform live cell measurements, and these setups typically depend on how the cells are confined, for example direct measurements of the sample onto a coverslip or CaF_2_ slide with a water immersion objective (Figure 6A) or using a sandwich method between two CaF_2_ slides (Figure 6B) or between a CaF_2 _and a coverslip with a coverslip compensated objective (Figure 6C). Recent efforts in live-cell O-PTIR have focused on enhancing spatial resolution, sensitivity, and temporal response to capture real-time biochemical changes. However, live-cell O-PTIR presents practical challenges, particularly the need for specialized environmental chambers that maintain cell viability while meeting stringent optical window requirements for simultaneous mid-IR excitation and visible photothermal detection.

To address these constraints, Paolone et al.^141^ developed an adaptable 3D-printed live-cell chamber compatible with multiple optical window materials. For Raman and O-PTIR measurements, cost-effective CaF_2_ windows were shown to be suitable, while zinc sulfide (ZnS), barium fluoride (BaF_2_), and diamond were also evaluated for extended spectral coverage. CaF_2_ and BaF_2_ exhibited high transmittance above 1300 and 1050 cm^−1^ (depending on the thickness), respectively, but their performance degraded at lower wavenumbers, limiting O-PTIR applicability in the far-IR region; BaF_2_, despite its improved low-frequency transmission, remains impractical due to its fragility and solubility in water. The chamber demonstrated airtight stability for over 15 days and enabled reproducible spectroscopic measurements, highlighting its potential for live-cell O-PTIR. Nevertheless, long-term use is likely constrained by the accumulation of metabolic gases and cellular byproducts, suggesting that practical live-cell measurements will be limited to significantly shorter durations.

In a notable study,^2^ FL-PTIR was applied to live green microalgae (putatively *Monoraphidium* spp.), enabling time-resolved hyperspectral imaging in water. A custom fluid cell was constructed using a thin adhesive spacer and sealed with a CaF_2_ window, allowing IR beam access while maintaining cell hydration. Using a 40× objective and a high numerical aperture setup, a hyperspectral image stack was acquired over 960–1800cm^–1^ with 4 cm^–1^ intervals and 0.4-second integration per frame. The data were compiled into a chemical imaging movie, capturing both spectral and spatial changes during cell motion. Although *Monoraphidium* is not typically motile,^142^ movement was observed, likely due to microfluidic currents or thermal gradients within the cell chamber demonstrating the feasibility of tracking live-cell chemical dynamics. However, if the identification is correct, the specific mechanism of the microalga motion is unknown. While no motion correction algorithms were applied, the study highlights the promise of FL-PTIR for real-time monitoring of live, hydrated microorganisms. Future implementations integrating particle tracking could further enhance spectral accuracy in dynamic systems.^143,144^

Recent advancements in O-PTIR-based metabolic imaging have shown promising potential for live-cell studies.^123^ A single-cell metabolic imaging platform utilizing azide-labelled palmitic acid enabled high-resolution, chemically specific visualization of lipid metabolism in fixed human iPSC-derived cells. While this work focused on fixed samples, the platform is readily adaptable to live-cell conditions. Prior studies have shown that spectral features in fixed and live cells are largely consistent, especially for key vibrational modes such as ester carbonyls and amide bands. Furthermore, thermal modelling estimates the local temperature rise during O-PTIR measurements to be less than 3 K and transient in duration, suggesting minimal interference with cellular physiology. These findings collectively support the feasibility of longitudinal, live-cell metabolic tracking with O-PTIR, opening avenues for real-time studies of lipid dynamics in health and disease.

Recent advances have demonstrated the power of O-PTIR in capturing lipid metabolic activity in live adipocytes with high spatial resolution.^124^ In particular, de novo lipogenesis (DNL) was visualized using azide-labelled precursors, enabling real-time tracking of lipid synthesis within and across individual cells. The study leveraged both hyperspectral imaging of fixed cells and single-frequency O-PTIR in live-cell mode, revealing consistent DNL rates across modalities. These results reinforce O-PTIR's utility for probing dynamic biochemical processes in physiologically relevant contexts.

To facilitate broader adoption of live-cell O-PTIR imaging, ongoing developments focus on reducing acquisition times while improving spatial and spectral performance. Advances such as widefield O-PTIR, fast-scanning QCL sources, and enhanced detector integration enable rapid, high-resolution imaging over larger fields of view, while advanced denoising and real-time signal processing improve sensitivity and limit light-induced thermal effects. These improvements expand the applicability of live-cell O-PTIR to time-resolved studies of cellular metabolism, subcellular dynamics, and disease progression.

In parallel, improved optics and laser sources have enabled detection of subtle biochemical changes, including protein folding, lipid metabolism, and nucleic acid interactions. Increasing integration of O-PTIR with complementary modalities such as fluorescence microscopy and Raman spectroscopy further enhances its utility by correlating chemical information with structural and functional readouts. Additionally, machine learning and artificial intelligence (AI)-based analysis tools are increasingly applied to live-cell O-PTIR datasets, enabling efficient interpretation of complex, high-dimensional data and providing deeper insights into dynamic processes such as cell signaling, gene expression, and metabolic regulation.

The advantage of live cell measurement comes with various considerations. Most of the challenges associated are explained in the Challenges and Limitations of Optical Photothermal Infrared Spectroscopy (O-PTIR) section, but in general, the measurements in aqueous environment are prone to thermal drift which may result in skewed results. As of right now, the instrument is not available with auto-tracking modality, which makes it difficult to acquire a large hyperspectral area scan of live cells. However, since single wavelength imaging is quick, live cell single wavelength imaging is possible.

### Integration with Artificial Intelligence and Machine Learning

The integration of AI and machine learning (ML) with O-PTIR is revolutionizing the field of chemical imaging. AI and ML techniques offer powerful tools for analyzing complex datasets, identifying patterns, and making predictions based on large volumes of data. In the context of O-PTIR, these technologies are being harnessed to enhance data acquisition, processing, and interpretation.

A key advancement in the field of O-PTIR is its integration with deep learning for tissue classification. In contrast to traditional machine learning techniques like random forests, convolutional neural networks (CNNs) have demonstrated exceptional performance in classifying^80^ O-PTIR hyperspectral images. In a recent large-scale study involving 78 ovarian cancer tissue samples, CNNs achieved an AUC of 0.98 for segmenting tissue regions, vastly outperforming traditional approaches. This performance gain is attributed to CNNs’ ability to simultaneously^58^ analyze spectral and spatial features in super-resolution data, an area previously unexplored in mid-IR imaging.

Despite computational challenges, including increased memory demands and alignment errors between tissue sections, the study provides a framework for balancing spatial and spectral resolution while maintaining classification accuracy. Furthermore, this analysis identified stromal and epithelial ratios (SR and ER) as potential quantitative biomarkers for early-stage ovarian cancer diagnosis. These results highlight the transformative potential of combining AI with O-PTIR for tissue-based diagnostics.

The synergy between O-PTIR spectroscopy and machine learning is opening new horizons in quantitative biomedical imaging. A compelling demonstration of this integration is in recent work applying CNNs and random forest (RF) algorithms to mid-infrared (MIR) photothermal imaging data for ovarian cancer tissue analysis.^145^ In this approach, high-dimensional hyperspectral data was processed to reconstruct spatially under sampled images using curvelet-based algorithms. Curvelet-based and deep learning-assisted reconstruction methods improve image sharpness and spatial fidelity from under sampled data, enabling accurate tissue differentiation with classification accuracies exceeding 95%, as validated by receiver operating characteristic curve (ROC) and quantitative metrics. AI-driven super-resolution and adaptive optimization of experimental parameters further enhance spatial resolution, data quality, and measurement reliability while minimizing sample damage. In addition, machine learning-based multimodal data fusion facilitates seamless integration of O-PTIR with Raman and fluorescence imaging, providing comprehensive chemical and structural insights. Collectively, these developments position AI-enabled O-PTIR as a key enabler of automated, high-resolution, and real-time biomedical imaging platforms.

### Interdisciplinary Innovations

Interdisciplinary innovations are driving the evolution of O-PTIR and expanding its applications across various fields. By combining O-PTIR with techniques from other disciplines, researchers are developing novel approaches to chemical imaging and enhancing the capabilities of O-PTIR.

The integration of O-PTIR with SERS represents a significant interdisciplinary advancement in vibrational spectroscopy. For instance, simultaneous O-PTIR and SERS imaging of colon cancer cells sorted by integrin activity enabled highly specific biochemical characterization of cellular subtypes.^58^ This dual-modality platform leverages the IR specificity and spatial fidelity of O-PTIR with the molecular selectivity of Raman, creating a powerful analytical tool for probing receptor dynamics, ligand interactions, and subcellular heterogeneity. Such innovations in hybrid systems underscore the evolving landscape of photothermal technologies in biomedical research.

Recent developments in atomic force microscopy infrared (AFM-IR) spectroscopy have demonstrated the potential of time-domain detection of photothermal signals using broadband femtosecond infrared pulses.^146^ By integrating a Michelson interferometer with peak force tapping AFM, FT-AFM-IR enables the acquisition of broadband IR spectra with nanoscale spatial resolution well beyond the conventional diffraction limit. A particularly intriguing finding in this approach is the observation of vertical asymmetry in photothermal interferograms, indicative of multiphoton vibrational excitation processes. Looking forward, further development of time-domain AFM-IR methods, such as incorporating controlled pulse sequences analogous to those used in 2D IR spectroscopy, promises to unlock unprecedented capabilities. These advances could enable nanoscale mapping of vibrational coherence and population dynamics, offering a transformative platform for chemical imaging with both ultrahigh spatial and spectral resolution.

Recent advances in nanoscale analytical methods have enabled detailed spectroscopic and microscopic investigations of individual substrate-deposited aerosol particles. Atomic force microscopy-based photothermal infrared spectroscopy (AFM-PTIR) uniquely combines the nanometer spatial resolution of AFM with the chemical specificity of vibrational infrared spectroscopy. In a notable demonstration, AFM-PTIR was applied to analyze both single- and multi-component aerosol particles composed of inorganic salts and organic compounds relevant to atmospheric chemistry. Remarkably, this technique enabled chemical and morphological characterization of particles as small as 50  nm in diameter. Furthermore, the study introduced,^147^ for the first time, single-particle AFM-PTIR measurements as a function of relative humidity. This capability allowed simultaneous and independent monitoring of photothermal IR spectra, contact resonance frequency shifts, and water uptake growth factors. Spectral data obtained from single-particle AFM-PTIR show good agreement with bulk FT-IR measurements, validating its reliability. Going forward, this technique promises to unravel localized chemical changes in multi-component aerosol particles subjected to reactive gas uptake and varying water vapor conditions, advancing our understanding of atmospheric particle behavior at unprecedented spatial resolution.

Total internal reflection photothermal detection spectroscopy (TIR-PTDS) has been demonstrated as a powerful approach for depth-selective infrared spectroscopy by modulating the thermal diffusion length (μS). Initial validation was performed on a simple phantom consisting of polymer foil and glucose layers on an internal reflection element (IRE). Extending this methodology to complex, multilayered biological systems such as skin revealed the potential of spectral depth profiling to selectively access IR information from distinct tissue depths. This depth-resolved capability significantly advances non-invasive glucose sensing by enabling selective measurement of IR spectra from glucose-relevant interstitial fluid layers within the skin.^148^ Recent advances in O-PTIR include electronically controlled double modulation of the quantum cascade laser, eliminating mechanical chopping and improving signal-to-noise ratio while enabling faster tuning and precise modulation synchronization. The extension of TIR-PTDS to IR spectral tomography enables depth-resolved analysis over tens of micrometers, benefiting biomedical applications such as skin characterization, drug penetration studies, and improved glucose monitoring, while also outperforming conventional ATR-IR for in situ analysis of multilayered industrial samples. An emerging multimodal approach, FISH-O-PTIR, integrates genetic localization with chemical imaging, allowing simultaneous visualization of gene-specific markers and molecular composition, and holds strong potential for advanced studies of gene expression and cellular heterogeneity.

### FISH-O-PTIR: Integrating Fluorescence In Situ Hybridization with O-PTIR for Single-Cell Metabolic Profiling

One of the most compelling interdisciplinary integrations involving O-PTIR is its combination with fluorescence in situ hybridization (FISH), resulting in the O-PTIR-FISH platform. This technique enables simultaneous identification of microbial species and analysis of their metabolic activity at the single-cell level, addressing the long-standing challenge of functionally profiling individual cells within complex microbial communities.

The core of O-PTIR-FISH lies in pairing rRNA-targeted FISH probes for cell identity with stable isotope labelling (e.g., ^13^C-glucose or D_2_O) to monitor metabolic flux. The incorporation of isotopic labels results in measurable red shifts in the IR absorption peak particularly in key vibrational modes such as the amide I band, allowing label-free, biochemical quantification of metabolic activity using O-PTIR.

In one study,^126^ researchers demonstrated the use of O-PTIR-FISH to track ^13^C assimilation in *E. coli* and mixed human gut microbiome samples. Despite the spectral complexity introduced by varied chemical compositions, the technique successfully distinguished labelled from unlabeled populations. The system was further validated using high-throughput image analysis, with quantitative metrics such as peak shift analysis and isotope content estimation across millions of data points.^125^

Beyond bacterial cultures, this method is adaptable to fungi and mammalian cells. For example, azide-labelled fatty acids can be used in lipid metabolism studies, while azidohomoalanine can track protein synthesis. This adaptability enables O-PTIR-FISH to probe a wide range of biomolecular processes (proteins, lipids, nucleic acids, carbohydrates) in different systems.

To enhance performance, future implementations may incorporate, widefield O-PTIR imaging for increased speed, machine learning–based denoising and image reconstruction and super-resolution IR imaging for improved spatial detail.

Despite minor challenges such as probe optimization and isotope detection limits in mixed populations, O-PTIR-FISH stands out as a powerful tool for vibrational imaging and quantitative, spatially resolved single-cell biochemistry. Its compatibility with established fluorescence workflows makes it highly accessible, paving the way for widespread adoption in microbiology, systems biology, and biomedical research.

## Challenges and Limitations of Optical Photothermal Infrared Spectroscopy (O-PTIR)

### Sample Preparation Challenges

Sample preparation for O-PTIR spectroscopy plays a crucial role in determining data quality, reproducibility, and interpretability. Although the technique is versatile accommodating a wide range of materials from synthetic polymers to complex biological specimens this flexibility also introduces challenges that must be addressed with careful experimental planning.

One of the primary complexities arises from the variability in sample types, each requiring a tailored approach. For example, thin polymer films may be analyzed with minimal preparation, whereas biological tissues or cultured cells typically demand sectioning, fixation, or embedding to maintain structural integrity and prevent drift during imaging. This is particularly important for live-cell measurements in aqueous environments, where thermal drift and sample instability are common. Ensuring adequate sample adhesion to the substrate is therefore critical to avoid movement during acquisition, especially over long imaging durations.

The choice of substrate is another significant consideration. While O-PTIR does not impose strict IR-transparency requirements as conventional transmission-based IR spectroscopy does, substrate selection still impacts spectral quality. Some of the aspect that should be paid attention during the sample preparation for O-PTIR are:

(i) Substrate Compatibility and IR Transparency: Unlike most of FT-IR spectroscopy, sample preparation for O-PTIR is not limited by the choice of substrate. Farnesi et al.^72^ have used glass as their substrate for cerumen sample analysis on O-PTIR and faced no interference from the background. According to the authors, glass slides are not the best substrate for their sample preparation, especially for thin samples <10  µm. There is a significant contribution from the background in the region from 1000–1200 cm^–1^ with the glass slide. The spectra consisting of glass signal is difficult to discriminate as itis a non-linear absorbance across the sample that depends on the sample thickness and the quality of glass used.
  The choice of substrate becomes critical, especially in co-propagation and counter propagation configurations. IR-transparent materials such as CaF_2_ are preferred but are fragile, expensive, and limited in size. All measurements in counter propagation mode are limited by the fact that sample must be opaque and preferably less than 10 μm in thickness^1^. Improper substrate selection can lead to spectral interference or signal attenuation, like in some cases Si as substrate turns out to be better than CaF_2_.^149^
(ii) Embedding and Fixation Artifacts: For tissue and cell samples, embedding media (e.g., paraffin, epoxy) and chemical fixatives (e.g., formaldehyde, glutaraldehyde) can introduce strong IR absorption bands or alter the native chemical structure of the sample. Cryosectioning and alcohol-based fixation (e.g., methanol, ethanol) may be an alternative, but it may still affect molecular conformation or hydration state, complicating spectral interpretation. For fine sectioning, microtomy can be preferred but in some cases, sectioning with surgical blade can also be a good option for observation.^1^
(iii) Sample Thickness and Uniformity: Sample thickness and uniformity are especially critical in O-PTIR, as the technique is sensitive to local heating effects that vary with sample morphology and thermal properties. Inhomogeneous or overly thick specimens can lead to signal distortion, saturation, or reduced spatial resolution. While techniques like spin coating^150^ and serial dilution (in drop casting) can be employed to regulate thickness, achieving consistent coverage is difficult particularly for heterogeneous or particulate samples.
(iv) Live Cell Imaging Constraints: Live-cell O-PTIR imaging requires maintaining cell viability while minimizing IR absorption by water. This necessitates specialized setups such as sealed fluid chambers or flow-through cells with controlled temperature, CO_2_, and nutrient supply. Additionally, the use of D_2_O instead of H_2_O is sometimes required to shift water absorption bands away from biologically relevant IR regions,^151^ but this can affect cell physiology.
(v) Fluorescence Labelling and Interference: Although O-PTIR is immune to fluorescence interference,^73,152^ the use of fluorescent labels must be carefully managed. Antifade reagents and some dyes contain IR-active components that can obscure key spectral features. Moreover, aligning fluorescence and IR images requires precise spatial registration, especially in multi-modal imaging workflows.
(vi) Sample Handling and Mounting: Mechanical stability during measurement is essential to avoid drift or misalignment. Biological samples, especially in aqueous environments, can shift or degrade over time. Mounting techniques must ensure both optical access and physical stability, which can be difficult when working with fragile IR windows or live specimens.

One of the primary challenges in sample preparation is ensuring that the sample is representative of the material being studied. This is particularly important in biological research, where heterogeneity within samples can lead to variability in the results. For example, when analyzing tissue samples, it is essential to obtain sections that accurately reflect the composition and structure of the tissue. This requires careful handling and processing to avoid artifacts that could skew the data. Finally, as O-PTIR continues to evolve towards multimodal and hyperspectral imaging, preparation protocols must accommodate the requirements of each analytical mode. For example, IR, Raman, and fluorescence imaging may each demand different optical properties, environmental conditions, or chemical treatments. Balancing these often-competing needs without compromising data integrity requires both technical skill and careful experimental design.

### Instrumental and Technical Constraints

While O-PTIR spectroscopy presents a promising avenue for high-resolution, label-free chemical imaging, several technical and instrumental limitations currently delay its broader adoption and routine use in biological and environmental studies.

A fundamental optical constraint in O-PTIR arises from its use of a visible probe laser, which determines the system's spatial resolution through visible-light diffraction limits. Unlike conventional FT-IR systems that employ broadband thermal sources such as globar, O-PTIR relies on tunable QCLs as mid-infrared (IR) excitation sources. This switch provides the advantage of narrowband, high-power IR radiation but simultaneously increases the overall energy deposited on the sample when combined with the visible probe. While the small sampling area enables sub-micrometer resolution and detailed analysis of limited sample volumes, it also results in a disproportionately high radiation dose relative to sample size. As a result, there is a heightened risk of sample degradation, particularly for heat-sensitive or soft biological materials. This necessitates careful optimization of laser power balancing the need for adequate signal strength against the risk of thermal damage.

Another significant limitation is the imaging speed, which is constrained by the use of single-element detectors. Unlike focal plane array (FPA) detectors employed in FT-IR or QCL-IR systems that enable rapid acquisition of large spectral maps, O-PTIR collects data in a point-by-point manner. This inherently limits throughput and makes hyperspectral imaging a time-consuming process. The spectral range is also confined to the tuning limits of the IR laser used standard QCLs typically cover ∼1800–800 cm^–1^, with some systems extending further through dual-laser (QCL-OPO) combinations, but this still does not match the broad spectral range of traditional FT-IR instruments.

The optical configuration of O-PTIR adds further complexity. Precise co-linear alignment of the IR pump and visible probe beams is essential for accurate detection of the photothermal signal. Any misalignment can result in diminished signal quality, reduced spatial resolution, or data artifacts. Additionally, system stability is highly sensitive to external factors such as temperature fluctuations, mechanical vibrations, and even slight optical drift necessitating frequent recalibration and a controlled laboratory environment.

The widefield imaging mode in O-PTIR presents an additional layer of intricacy. This mode is limited to counter-propagating geometries, requiring specific sample preparation protocols that differ from those used in co-propagated setups. Furthermore, widefield O-PTIR measurements rely on changes in fluorescence intensity rather than direct IR absorption, making quantitative interpretation more challenging. The signal can be influenced by the laser beam profile, which may not be perfectly uniform. As a result, uneven illumination across the field of view can degrade image quality. In addition, fluorescence is inherently prone to quenching, especially under high laser intensities even when defocused. While widefield images remain usable due to the relative nature of fluorescence changes, absolute quantification is difficult, and care must be taken to avoid signal degradation due to photobleaching or quenching effects.

Instrument cost is another practical barrier. Due to the need for high-quality, tunable laser sources, sensitive detectors, and robust optical alignment systems, O-PTIR instruments remain expensive and are not yet widely available. This limits accessibility, particularly for laboratories without specialized photothermal spectroscopy infrastructure. The lack of standardized operating protocols further hampers reproducibility and cross-laboratory comparability.

In biological applications, additional constraints have become evident. For instance, the inherent curvature of cells, such as the biconcave shape of red blood cells (RBCs), can lead to optical artifacts. Unlike Raman systems equipped with features like “true surface mapping” (e.g., those by WITec), which can optically correct for surface topology, O-PTIR lacks native curvature correction. This limitation can result in imaging distortions such as hollow center's or signal shadows in curved structures, where changes in geometry interfere with accurate chemical interpretation.

Environmental control is another critical consideration. Biological samples are highly sensitive to humidity fluctuations, which can introduce interfering water vapor bands in the IR spectrum. Hence, O-PTIR measurements should ideally be conducted in a nitrogen-purged chamber, with humidity levels maintained below 20%. It is advisable to initiate nitrogen flow at least 30–60 minutes prior to sample placement to ensure a stable environment.

Furthermore, O-PTIR's reliance on Cassegrain objectives necessary for co-propagated IR and visible beam paths limits its compatibility with powdered or loosely bound samples. These objectives use reflective optics and introduce airflow within the optics chamber, which may cause lightweight particles to be displaced due to positive pressure, complicating measurements of such materials. Also, Cassegrain objectives are in general not very good from the resolution point of view, which is not a problem in IR spectra, but it cost heavily on Raman spectroscopy.

Despite offering excellent IR spatial resolution, O-PTIR faces limitations in Raman resolution and acquisition speed. Hyperspectral image acquisition is particularly time-intensive, with measurements affected by sample drift, non-uniformity, and laser-induced stress or damage. While integrating multimodal imaging such as Raman, fluorescence, and polarization techniques enhances analytical depth, it also increases instrumental and computational complexity. Ensuring all modalities operate synergistically requires meticulous calibration and dedicated software configurations.

A notable limitation of O-PTIR lies in its challenges for precise quantitative analysis of component concentrations. Unlike traditional FT-IR spectroscopy, which relies on the Beer–Lambert law for direct quantification through absorbance measurements, the O-PTIR signal, while linearly related to concentration and molecular absorptivity, is also influenced by additional factors such as the sample's size in a more complex manner. Furthermore, physical properties of the material including density, heat capacity, and thermal conductivity affect the O-PTIR response and are not considered in the Beer–Lambert framework. Due to these complexities, absolute signal amplitude from O-PTIR is typically not employed for direct quantitation. Instead, semi-quantitative assessments are made using ratiometric techniques, like comparing intensity ratios of different spectral features or absorption at multiple wavelengths. For example, the ratio between a carbonyl ester absorption band and an amide band can be used to estimate relative variations in lipid and protein concentrations.^1^ These ratiometric methods provide a practical workaround for some quantification challenges in O-PTIR, though fully absolute concentration determination remains difficult.

Finally, although single-point detectors are the standard in current O-PTIR setups, their slow acquisition rates pose a bottleneck. While using multi-element detectors like focal plane arrays (FPA) could theoretically alleviate this limitation, doing so would significantly increase the instrument's cost and complexity already a notable concern given the high baseline price of existing systems.

Optical photothermal infrared spectroscopy offers significant advantages in terms of spatial resolution and non-contact infrared spectroscopy, it demands a high level of technical expertise, strict environmental control, and careful sample preparation. Addressing these technical and instrumental constraints will be essential to realize its full potential in routine, large-scale, or automated analytical workflows.

### Complexity in Data Interpretation

One of the key challenges associated with O-PTIR spectroscopy is the complexity involved in accurately interpreting the acquired data. Unlike conventional IR spectroscopy, where signal intensity is largely determined by molecular absorption, the O-PTIR signal is modulated by a broader set of sample-dependent physical and thermal properties. These include parameters such as sample thickness, distance from the focal plane, heat capacity, thermal conductivity, density, and particle size. Together, these factors influence the degree of local heating and cooling following IR excitation, thereby affecting the amplitude and distribution of the photothermal signal. As a result, contrast in single-wavenumber O-PTIR images may not correspond solely to chemical absorption, making straightforward interpretation more difficult.

To mitigate these challenges, two advanced imaging strategies are commonly adopted: ratiometric imaging and multi-color overlays. Ratiometric imaging involves acquiring O-PTIR signals at two or more wavenumbers and computing their intensity ratios. This technique can help normalize non-absorptive effects such as variations in thermal properties or surface reflectivity by assuming that these remain relatively constant across the selected bands. The resulting ratio images more accurately reflect chemical composition and are especially valuable when acquired in an interleaved fashion to minimize drift. To avoid noise amplification or division artifacts, appropriate signal thresholds must also be applied during processing.

**Figure 7.**
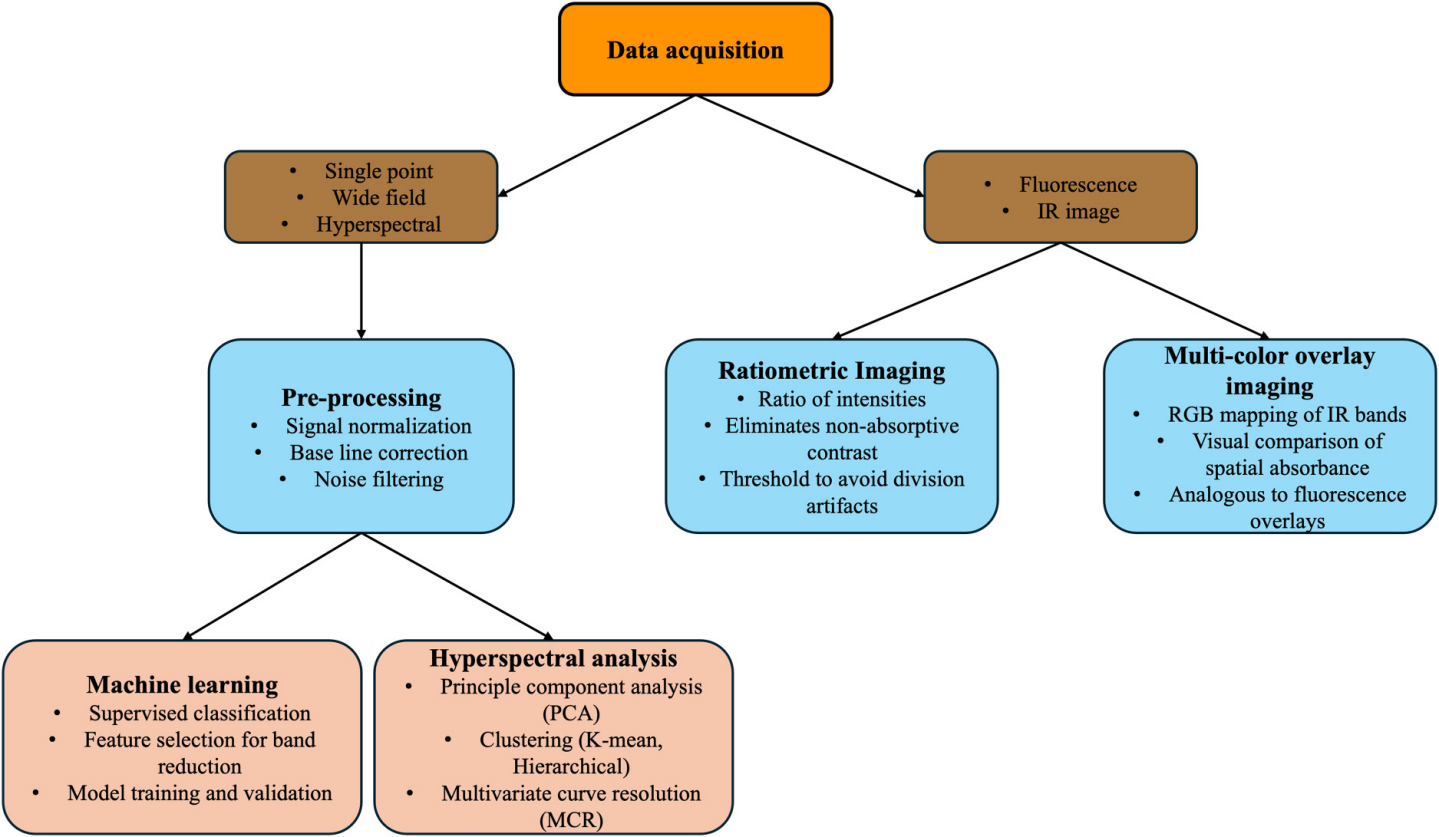
Generalized data processing pipeline for O-PTIR data.

Multi-color overlay imaging, by contrast, offers a more qualitative approach. By assigning different colors to different IR absorption bands, like how fluorescence channels are visualized, researchers can highlight spatial variations in chemical distribution within heterogeneous samples. While this method does not correct for thermal or optical artifacts, it provides a highly intuitive means of identifying regions of interest, particularly in complex biological systems. Figure 7 shows various settings associated with the data interpretation in O-PTIR.

With the growing capability of O-PTIR systems to acquire hyperspectral datasets either full spectra per pixel or sequential band-specific images the demand for robust data analysis tools has increased significantly. Analyzing such high-dimensional data often requires multivariate statistical approaches or machine learning methods. Software platforms such as CytoSpec, Eigenvector Research, and the open-source Quasar platform^82^ provide functionalities for techniques like principal component analysis (PCA), hierarchical and *k*-means clustering, and multivariate curve resolution (MCR). These tools are instrumental in isolating chemically distinct domains, identifying spectral outliers, and improving tissue classification and diagnosis.

Moreover, supervised learning models are being increasingly utilized to optimize both data acquisition and interpretation. Recent studies demonstrate that deep learning algorithms can identify the most informative wavenumbers for classification, enabling faster and more computationally efficient imaging without sacrificing accuracy. This is particularly promising for clinical diagnostics and other high-throughput applications, where speed and reproducibility are critical.

One of the challenges in interpreting O-PTIR data arises from the dual-laser setup required for simultaneous acquisition of infrared and Raman spectra. The high energy of both the pump (IR) and probe (visible) lasers can locally heat the sample, potentially leading to thermal effects such as spectral distortion or even degradation of biological cells. Lima et al.^3^ addressed this concern by systematically acquiring 50 Raman and infrared spectra from the same spot on both a single bacterium and a bulk bacterial population. This approach was used to assess how repeated laser exposure might influence spectral quality. PCA of these spectra revealed important trends. For infrared spectra, the first 10 acquisitions showed minimal changes, indicating negligible thermal impact during short scans. However, when 50 spectra were collected, a clear trend emerged along the principal component (PC-1) axis, suggesting spectral shifts potentially due to cumulative thermal effects. This trend was observed in both single-cell and bulk measurements. Raman spectra, on the other hand, showed more immediate and pronounced changes even within the first 10 scans highlighting their greater sensitivity to laser-induced heating. These spectral variations raise concerns about the potential for thermal artifacts, especially when analyzing subtle biochemical differences or when using multivariate statistical tools like PCA, which are sensitive to even small spectral changes. Despite this, Lima et al.^3^ emphasized that the thermal effects observed were minor compared to the significant spectral changes caused by the incorporation of heavy isotopes (^13^C and ^15^N), which dominated the PCA interpretation. Nonetheless, their study highlights the need for caution. Misinterpretation or misclassification can easily occur if thermal artifacts are not properly accounted for especially in high-sensitivity analyses using O-PTIR.

There is also increasing interest in applying O-PTIR for surface-enhanced techniques, such as single-molecule detection using SERS and surface-enhanced infrared absorption (SEIRA). However, quantitative interpretation of photothermal signals in such cases remains non-trivial. The signal response is governed by complex, non-linear interactions between refractive index changes and local thermal dynamics and does not follow a simple linear correlation with analyte concentration. As such, more sophisticated calibration and modelling strategies are often required to extract meaningful quantitative data.

Additionally, one must be aware of instrumental artifacts associated with the use of QCL sources. Each QCL chip has a characteristic spectral drop-off near its tuning edge, which can introduce artificial sharp features resembling spectral peaks. Although this can be corrected through optimization in software or post-processing, researchers should remain cautious not to misinterpret such artifacts as genuine spectral features, particularly when stitching together spectra across different QCL modules.

Optical photothermal infrared spectroscopy enables highly detailed spatial and spectral imaging, the complexity of its signal formation, susceptibility to sample-specific variables, and potential for optical artifacts present considerable analytical challenges. Successful application of this technique requires not only careful experimental design but also advanced data processing and interpretation workflows. Continued development of robust analytical algorithms and artifact correction strategies will be essential to fully unlock the technique's potential in both research and clinical settings.

## Conclusion

Optical photothermal infrared spectroscopy (O-PTIR) has emerged as a powerful and transformative vibrational spectroscopy technique by integrating the molecular specificity of infrared absorption with the high spatial resolution of visible-light detection. By exploiting the photothermal effect, O-PTIR overcomes the diffraction limit of conventional IR spectroscopy, enabling sub-micrometer-scale, label-free, and minimally invasive chemical imaging. The flexibility of its experimental configurations, provide a truly multimodal platform for comprehensive chemical and structural analysis.

Compared with traditional FT-IR and Raman techniques, O-PTIR offers superior spatial resolution, compatibility with standard sample formats, and enhanced sensitivity, making it particularly well suited for complex biological systems. Its applications span cellular and subcellular imaging, microbial identification, tissue diagnostics, and pharmaceutical research, enabling detection of biomolecules, monitoring of metabolic processes, identification of disease-related biomarkers, and investigation of drug–cell interactions with unprecedented detail. These capabilities are especially impactful for live-cell and native-state studies, where preserving biological integrity is critical.

Despite its significant advantages, challenges related to sample preparation, instrumental complexity, and data interpretation remain and warrant continued methodological and analytical development. Looking forward, advances in live-cell O-PTIR, integration with machine learning, and emerging hybrid approaches such as FISH-O-PTIR is expected to further expand its analytical power. As these developments mature, O-PTIR is destined to play an increasingly central role in biological and biomedical research, driving new insights into molecular processes and enabling innovative diagnostic and therapeutic strategies.
